# A Multistate Adaptive System of Topologically Distinct Chiral Assemblies

**DOI:** 10.1002/anie.202509903

**Published:** 2025-07-11

**Authors:** Wiktoria Adamska, Grzegorz Markiewicz, Anna Walczak, Gokay Avci, Kim E. Jelfs, Jeremy K. M. Sanders, Artur R. Stefankiewicz

**Affiliations:** ^1^ Center for Advanced Technologies Adam Mickiewicz University in Poznań Uniwersytetu Poznańskiego 10 Poznań 61–614 Poland; ^2^ Faculty of Chemistry Adam Mickiewicz University in Poznań Uniwersytetu Poznańskiego 8 Poznań 61–614 Poland; ^3^ Department of Chemistry, Molecular Science Research Hub White City Campus, Imperial College London Wood Lane London W12 0BZ UK; ^4^ Yusuf Hamied Department of Chemistry University of Cambridge Cambridge CB2 1EW UK

**Keywords:** Guest binding and release, Nanotubes, NDIs, Non‐covalent interactions, Supramolecular polymerization

## Abstract

Biological systems exemplify the extraordinary adaptability of living organisms to their surroundings, demonstrating the capacity to reconfigure their structure, properties, and function in response to specific environmental signals. In this context, the exploration of multistate chemical systems designed to mimic natural counterparts, with the capacity to precisely tailor the structural outcome of an assembly and perform a specific function in response to external triggers, remains largely unexplored. Herein, we present a multistate adaptive system of topologically distinct chiral assemblies obtained from a single amino acid‐derived naphthalene diimide component and demonstrate its trigger‐responsive properties. Our system yields five distinct supramolecular assemblies across both solution and solid states, achieved by modulation of external factors such as temperature, solvent, concentration, and guest molecules. The work demonstrates the remarkable adaptability of the non‐covalent assemblies, revealing their profound sensitivity to external triggers, emphasizing the role of enthalpy and entropy in navigating across complex assembly pathways to and between individual outcomes.

## Graphical abstract

This article presents a non‐covalent multistate system with seven distinct assembly products across solid and liquid states, generated from a single supramolecular synthon. Thermodynamic pathways between these states are switchable with simple external triggers. This system could pave the way for new smart materials and offer fresh insights into molecular dynamics by imitating the flexibility of biological systems.

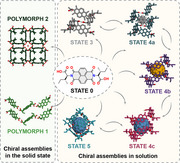

## Introduction

Naturally occurring systems exhibit exceptional versatility, seamlessly altering their structure, properties, and functionality in response to changing environmental factors, highlighting their multi‐responsiveness and adaptive nature.^[^ ^1^, ^2^, ^3^ ^]^ A classic example of this is DNA, which undergoes denaturation and renaturation in response to temperature changes, thereby influencing fundamental processes such as replication and transcription.^[^ ^4^, ^5^ ^]^ Another fascinating example is the pine cone, which cleverly responds to ambient humidity by opening its flaps to release seeds when growth conditions are ideal. This activation is triggered by the swelling of a specific hygroscopic layer due to moisture, resulting in a unique anisotropic response.^[^ ^6^ ^]^ Chemists have shown a growing interest in creating multi‐trigger‐responsive and adaptive assemblies that mimic naturally existing biological structures.^[^ ^7^, ^8^, ^9^, ^10^, ^11^, ^12^, ^13^, ^14^ ^]^ However, artificial systems, particularly non‐covalent ones, often struggle to match the complexity of their natural counterparts, as they typically operate between only two distinct states, consequently limiting their functional diversity.^[^ ^15^, ^16^, ^17^, ^18^, ^19^, ^20^, ^21^ ^]^ Addressing this limitation, the development of functional multistate systems with inherent chirality and binding capabilities represents a considerable challenge.^[^ ^22^, ^23^, ^24^, ^25^, ^26^, ^27^, ^28^, ^29^, ^30^, ^31^ ^]^ One promising avenue lies in designing chiral components capable of undergoing conformational changes at both the monomeric and supramolecular levels.^[^ ^22^, ^32^ ^]^ Notably, a group meeting these criteria is the amino‐acid naphthalene diimide (NDI) derivatives, known for their diverse self‐assembly capabilities.^[^ ^22^, ^23^, ^33^, ^34^, ^35^, ^36^, ^37^ ^]^

Unlike most of the supramolecular systems, which are typically bi‐stable,^[^ ^38^, ^39^, ^40^ ^]^ our approach enables the reorganization of a single component into five distinct supramolecular assemblies, each with individual topology, chirality, and stability. This diversity ranges from *quasi*‐1D and *quasi*‐2D polymers through nanotubes^[^ ^34^ ^]^ to capsules,^[^ ^36^ ^]^ with functional properties that dynamically adapt to external environmental triggers. The primary novelty of this system lies in its highly adjustable and reversible nature, accomplished by steering the system with external stimuli such as temperature, concentration, solvent type or guest molecule interactions, allowing transitions between specific states under complex dynamic equilibria. These equilibria significantly impact the functionality of the resulting assemblies. The adaptive and diverse nature of this system is exemplified by the selective identification of guest molecules in specific states. Energetic analysis supported by Density Functional Theory (DFT) calculations has provided crucial insights into the molecular mechanisms, deepening our understanding of environmental influences and enabling precise control over the structural and functional evolution of such multistate adaptive systems.

## Results and Discussion

**Figure 1:**
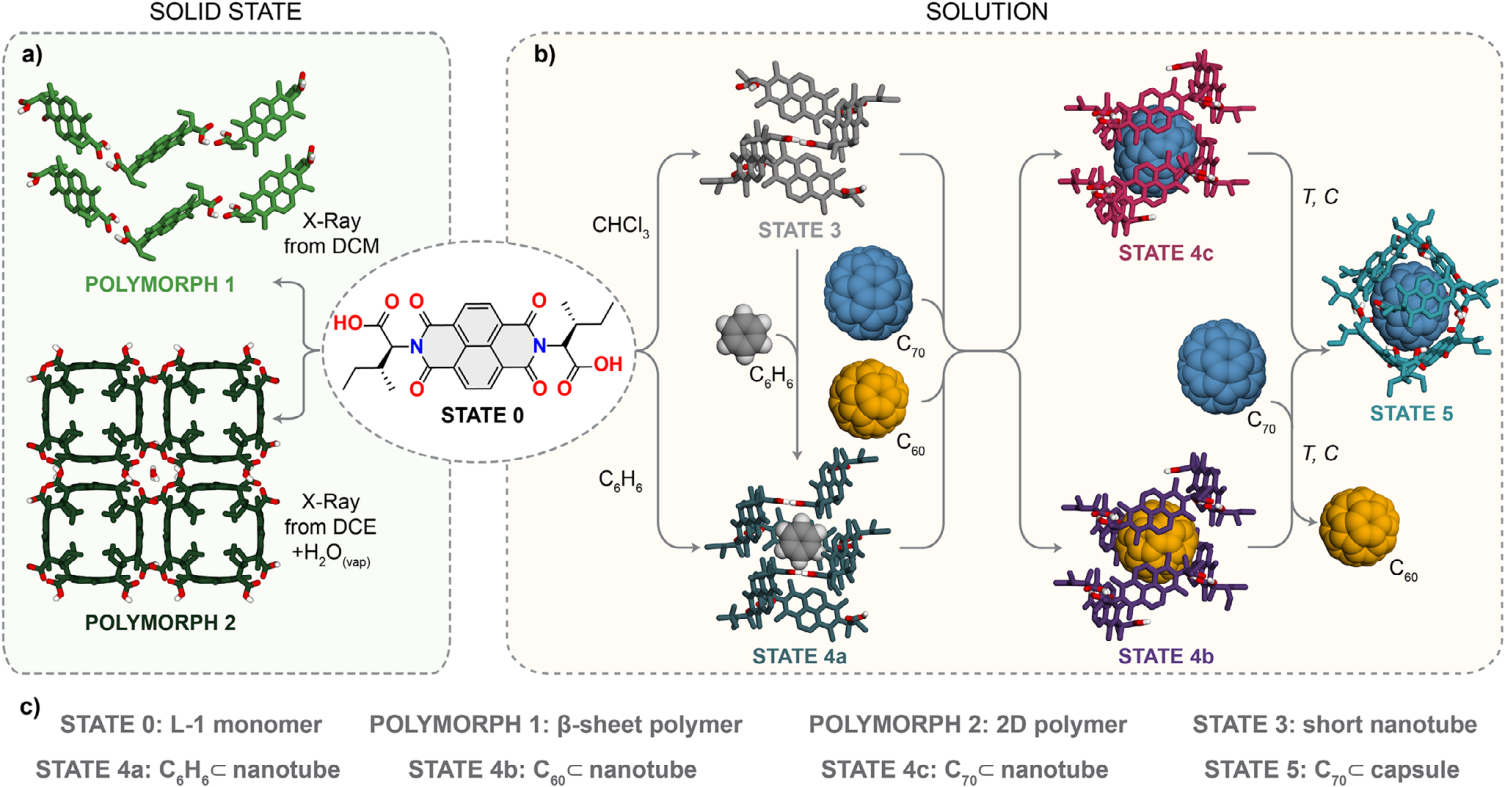
a) Chemical structure of l‐ **1** and X‐ray structures of its polymorphs observed in the solid state. b) Chemical structure of l‐**1** and proposed DFT models of its specific states in solution. A structural reorganization of this artificial system into five distinct supramolecular states was accomplished, through modulation of solvent, temperature, concentration, and guest molecules. c) The nomenclature of polymorphs and states.

At the outset of these studies, we hypothesized the pivotal role of the l‐isoleucine derivative of 1,4,5,8‐naphthalene‐tetracarboxylic dianhydride (l‐**1**) in establishing a versatile, multistate supramolecular system. Our vision involved leveraging the synergistic interactions of this component with various sets of reaction conditions and guest molecules, thereby unlocking a diverse array of potential transformations. This versatility results from the capacity of amino acid‐functionalized NDI building blocks to assemble into distinct aggregates through a range of supramolecular interactions (e.g., H–bonding or π–π stacking), involving different functional groups inherent within the component.^[^ ^33^, ^41^ ^]^ To explore this potential, we synthesized l‐**1**, (STATE 0) under previously described conditions (See Supporting Information for details).^[^ ^42^ ^]^ Initially, we performed X‐ray structure determination on the crystals of l‐**1** (Figure 1a on the left). Two distinct polymorphs of l‐**1** (Polymorph 1 and 2) were obtained by the spontaneous evaporation of its dichloromethane (DCM) or 1,2‐dichloroethane (DCE) solutions. In solution, however, the same compound assembled into three additional distinct supramolecular aggregate states, depending on the triggers and conditions employed (Figure 1 on the right).

### Polymorph 1–β‐sheet‐Like Polymer

To generate crystals corresponding to Polymorph 1,^[^ ^43^ ^]^ the l‐**1** was dissolved in DCM, and the solvent was allowed to evaporate at ambient conditions. The resulting crystals revealed the formation of the l‐**1** × ⅓ CH_2_Cl_2_ solvate, adopting a β‐sheet‐like supramolecular polymer configuration.^[^ ^33^ ^]^ In this assembly, the amino acid side chains adopted an *anti*‐conformation around the NDI plane, with the individual molecules of l‐**1** connected via hydrogen bonding between carboxylic units (OH^…^O═C), giving rise to a single‐stranded 1D polymer (schematically shown in Figure 1a, top). Furthermore, the individual strands formed connections via CH^…^O/O^…^HC side‐to‐side interactions between NDI units, ultimately yielding a β‐sheet‐like aggregate (a more detailed description is available in Section 4 of the SI).

### Polymorph 2–2D Polymer

When l‐**1** was dissolved in DCE, an entirely different aggregate emerged. It is noteworthy that DCE, being substantially less volatile than DCM, resulted in a prolonged evaporation under ambient conditions. This allowed enough time for the solution to saturate with water vapour, eventually leading to the formation of crystals corresponding to l‐**1** hydrate,^[^ ^43^ ^]^ denoted as (l‐**1**)_4_ × H_2_O in Polymorph 2. This assembly involves polymers generated by carboxyl group interactions and separated by sheets of water molecules (schematically shown in Figure 1a, bottom). Both polymorphs of l‐**1** in the solid state highlight that, although OH^…^O═C interactions may be considered as potentially the strongest force in operation,^[^ ^44^ ^]^ they can manifest in different forms that ultimately balance various other weak interactions. For instance, the direct, double OH^…^O═C bonding of carboxylic acid units observed in Polymorph 1 is compatible with extended CH^…^O interactions of the NDI units. On the other hand, the bridging of two carboxylic acid groups by another in Polymorph 2, is compatible with the stacking of the NDI units (a more detailed description is available in Section 4 of the SI).

### State 3–Short Nanotube

**Figure 2:**
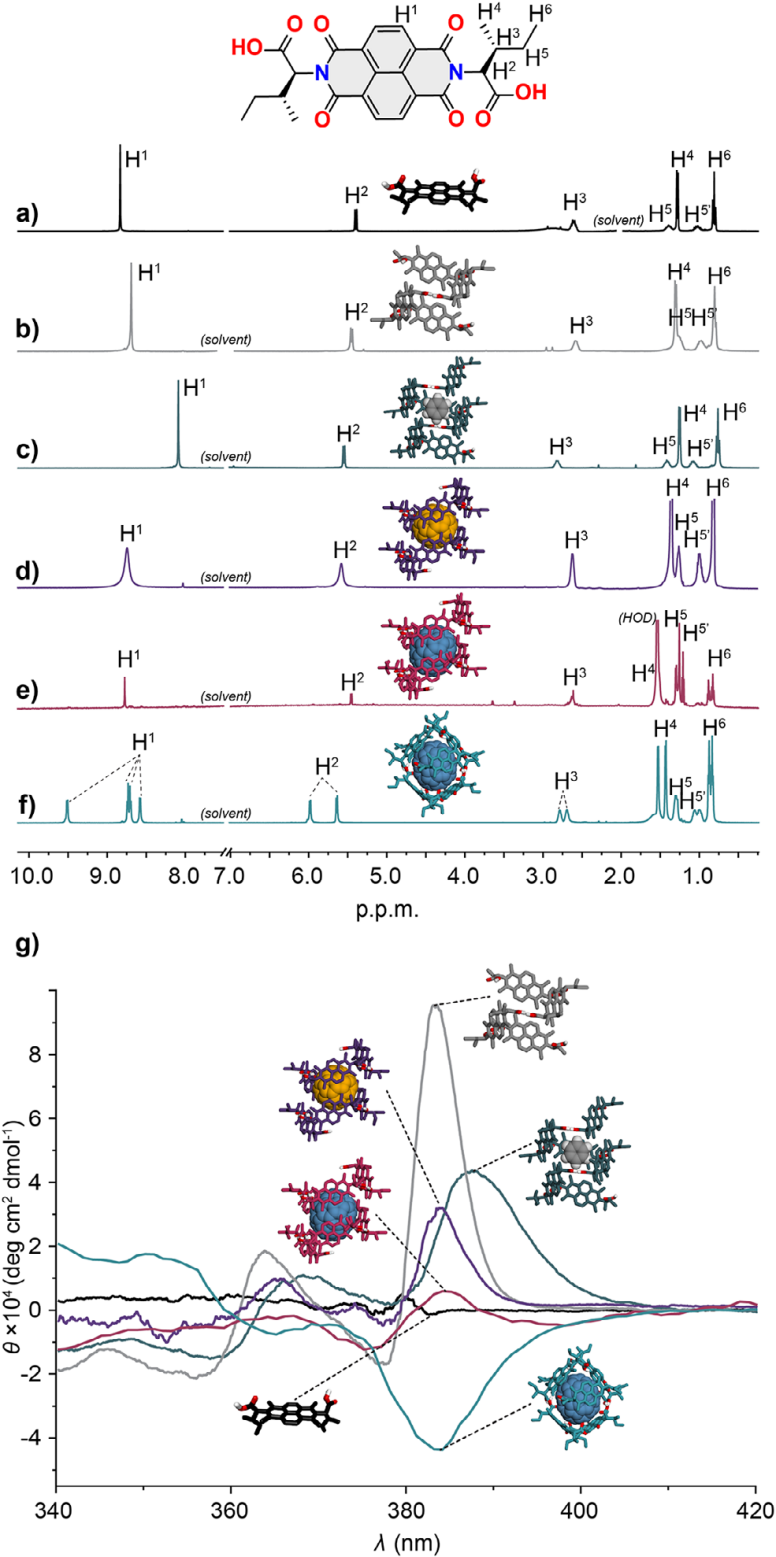
^1^H NMR spectra (600 MHz, *T* = 298 K) of l‐**1** in: a) (CD_3_)_2_CO, *C_NDI _* =* *1.0 × 10^−2^ m; b) CDCl_3_, *C_NDI _* =* *1.0* *×* *10^−2^ m; c) C_6_D_6_, *C_NDI_* =* *1.0 × 10^−2^ m; d) CDCl_3_ with C_60_, *C_NDI_* = 1.0 × 10^−2^ m; e) CDCl_3_ with C_70_, *C_NDI_* = 1.0 × 10^−4^ m; f) CDCl_3_ with C_70_ *C_NDI_* = 1.0 × 10^−2^ m. g) Normalized CD spectra *(T* = 298 K) of: STATE 0: (CH_3_)_2_CO, *C_NDI_* = 1.0 × 10^−3^ m; STATE 3: CHCl_3_, *C_NDI_* = 1.0 × 10^−3^ m; STATE 4a: C_6_H_6_, *C_NDI_* = 1.0 × 10^−3^ m; STATE 4b: CHCl_3_, *C_NDI_* = 1.0 × 10^−3^ m; STATE 4c: CHCl_3_, *C_NDI_* = 1.0 × 10^−4^ m; STATE 5: CHCl_3_, *C_NDI_* = 1.0 × 10^−2^ m.

The self‐assembly of l‐**1** in chloroform (CDCl_3_), a low‐polarity and non‐competitive solvent, leads to the formation of short hydrogen‐bonded assemblies, as confirmed by NMR and FT‐IR spectroscopies (for a detailed analysis see Section 5 in Supporting Information). The ^1^H NMR spectrum at 298 K in CDCl_3_, shows that l‐**1** preserves its inherent twofold symmetry in this solvent, as indicated by a single set of resonances (Figure 2b). The NDI proton (8.70 p.p.m.) undergoes an upfield shift (0.1 p.p.m.), while the α‐CH signal (5.45 p.p.m.) shifts downfield (by 0.1 p.p.m.), relative to the spectrum of l‐**1** recorded in acetone ((CD_3_)_2_CO), where hydrogen bonding is restricted to solvent‐solute interactions (Figures 2a and S6a). The ^13^C NMR and FT‐IR spectra provide further evidence for the formation of COOH^…^HOOC bridges, as the COOH resonance shifts downfield by approximately 5 p.p.m. (Figure S6b), and the *ν*C═O band of the COOH group undergoes a bathochromic shift from 1750 to 1725 cm^−1^, respectively (Figure S7a). The formation of single species in CDCl_3_ was further confirmed by DOSY‐NMR, with all the signals displaying the same diffusion coefficient (Figure S10b), corresponding to a solvodynamic radius *r*  = 0.76 nm (as oppose to 0.43 nm in (CD_3_)_2_CO, Figure S10a), consistent with the generation of an ensemble of relatively short polymeric species (Figure S12), averaged on the NMR‐diffusion time scale. These findings are in agreement with the formation of COOH^…^HOOC and CH^…^O hydrogen bonds between l‐**1** molecules, leading to the formation of short helical nanotubes (Figure 1b, STATE 3), similar to those observed both in solution and in the solid state for an analogous NDI derivative.^[^ ^34^ ^]^

**Figure 3:**
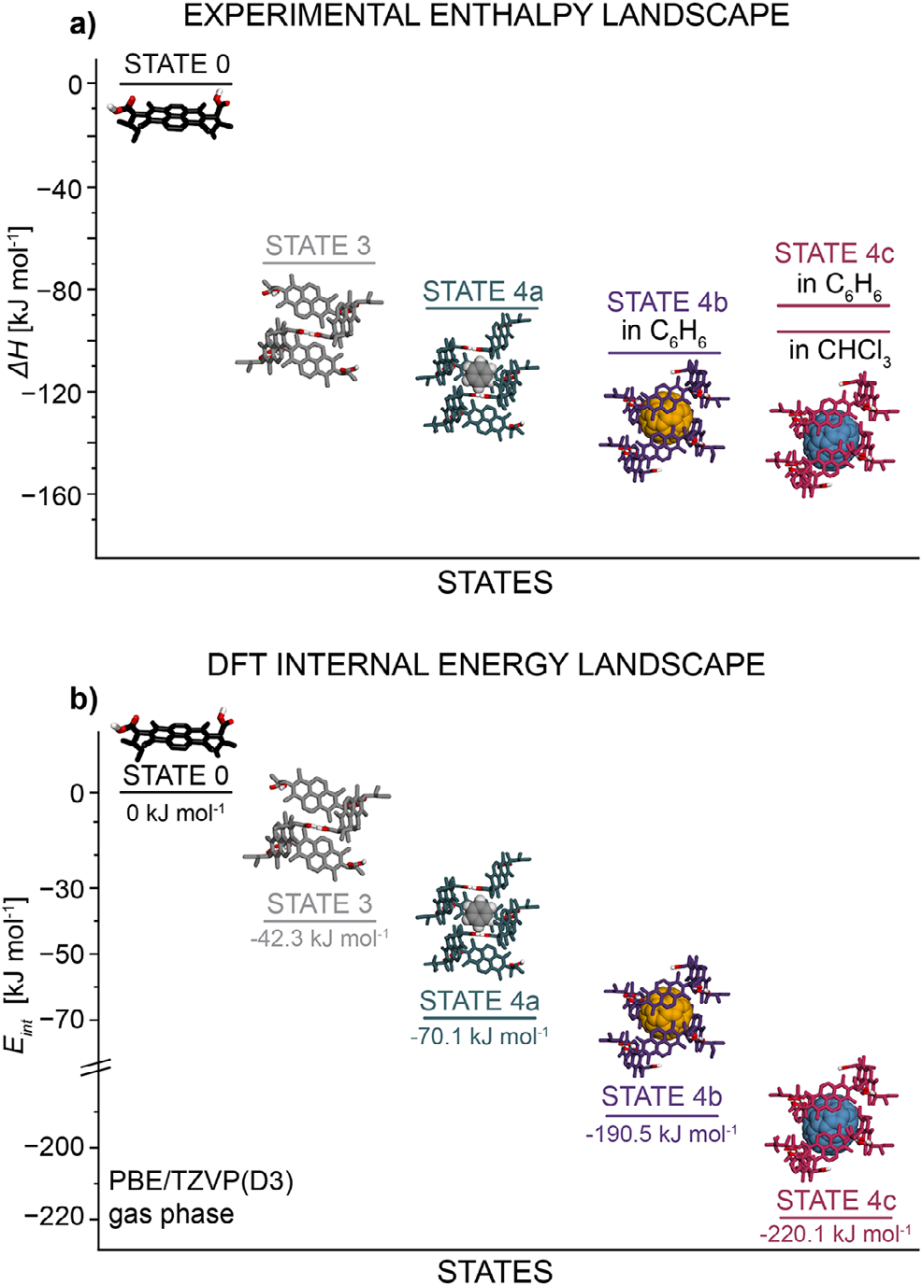
a) Experimental enthalpy landscape obtained from VT‐CD experiments in CHCl_3_ or C_6_H_6_ solutions. b) Theoretical (DFT) internal energy landscape (gas phase).

**Figure 4:**
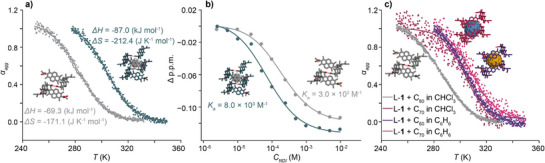
a) Temperature‐dependent CD intensity plots recorded during cooling of the solutions of l‐**1** in CDCl_3_ (grey trace) and C_6_D_6_ (teal trace) *C_NDI_* = 1.0 × 10^−4^ m, *T* = 340–250 K, cooling rate −1 K min^−1^. b) Concentration‐dependent ^1^H NMR chemical shifts of l‐**1** recorded in CDCl_3_ (grey trace) and C_6_D_6_ (teal trace). c) Temperature‐dependent CD intensity plots recorded during cooling (−1 K min^−1^) of solutions of l‐**1** (*C_NDI_ *= 1.0 × 10^−4^ m) with C_60_ and C_70_ in CHCl_3_ and C_6_H_6_.

Computational modeling of STATE 3 (Figure 3b) revealed that this assembly is indeed held together by COOH^…^HOOC hydrogen bonding between l‐**1** units, and the thus formed chain coils into an infinite helical structure thanks to the *syn*‐conformation around NDI‐plane and supplementary CH^…^O interactions (a more detailed description is provided in Section 8 of the SI, with energy values in Table S2). The formation of STATE 3 represents the 1^st^ step on the energy landscape ladder (Figure 3a,b), with the calculated drop in *E_int_* of −42 kJ mol^−1^ (PBE/TZVP(D3) DFT‐ gas phase), in agreement with the experimentally observed enthalpy release of *ΔH* = −69.3 kJ mol^−1^, and free energy release of *ΔG_298 K_* = −18.3 kJ mol^−1^ determined from variable temperature circular dichroism (VT‐CD) experiments (Figure 4a, grey trace). Although the simulations were performed in the gas phase and do not account for solvation, the qualitative trends from the simulations show correspondence with experimental thermodynamic parameters such as enthalpy.

The sigmoidal changes in CD intensity as a function of temperature indicated thermodynamically‐controlled isodesmic growth, while the negative enthalpy change confirmed that the process is driven by the energy of the newly formed hydrogen bonds. Further evidence for the thermodynamically controlled self‐assembly came from the concentration‐dependent ^1^H NMR experiments (*T* = 298 K), where changes in the chemical shift of NDI resonance as a function of *logC_NDI_* revealed a clearly sigmoidal (isodesmic) transition, with *K_a_* = 3.0 ± 0.2 × 10^3^ m ^−1^ (Figure 4b, grey trace).

### State 4a–C_6_D_6_@Nanotube

STATE 4a can be achieved by interacting l‐**1** with benzene (C_6_D_6_), a solvent that has lower polarity and distinct host:guest properties compared to CDCl_3_, which was used to obtain STATE 3. Analysis through ^1^H, ^13^C NMR, and FT‐IR spectra confirmed the formation of a helical nanotube in C_6_D_6_ (for a detailed analysis see Section 5 in Supporting Information), similar to the STATE 3 but notably longer and possessing enhanced thermodynamic stability (vide infra).

The ^1^H NMR spectrum in C_6_D_6_ (Figures 2c and S6) revealed a more pronounced upfield shift of the NDI resonance by ≈0.6 p.p.m. compared to a solvated monomer, indicative of interactions extending beyond CH^…^O hydrogen bonding to involve π–π interactions between the aromatic units. DOSY NMR analysis, performed at the same conditions as for STATE 3, revealed a substantial increase in the solvodynamic radius of the STATE 4a, reaching 0.94 nm (Figure S10c), again indicating the presence of an ensemble of polymeric species (Figure S12), averaged on the NMR‐diffusion time scale.

The formation of STATE 4a constitutes the 2nd step on the energy landscape ladder, located 18 kJ mol^−1^ below STATE 3 in terms of *ΔH* and 5 kJ mol^−1^ in terms of *ΔG_298 K_* (Figure 3a). Variable temperature CD experiments, performed at the same conditions as for STATE 3, again indicated thermodynamically controlled isodesmic growth with an enthalpy release of *ΔH *= −87.0 kJ mol^−1^ and free energy release of *ΔG_298 K_ = *−23.6 kJ mol^−1^ (Figure 4a, teal trace). The higher enthalpy release in C_6_H_6_, along with a slightly higher entropic penalty (*ΔS* = −212 J K mol^−1^ in C_6_H_6_, *ΔS* = −171 J K mol^−1^ in CHCl_3_), resulted in greater degrees of aggregation under the same *C_T_* and *T* conditions, aligning with DOSY results (Figure S10c). Additionally, concentration‐dependent ^1^H NMR spectra (*T *= 298 K, Figure 4b, teal trace) confirmed an isodesmic polymer growth, with *K_a_* = 8.0 ± 1.0 × 10^3^ m ^−1^, corroborating the higher degree of aggregation of l‐**1** in C_6_D_6_ compared to CDCl_3_.

The observed difference in the apparent thermodynamic stability between STATEs 3 and 4a could be attributed to various factors. This difference may arise from additional intermolecular host:guest‐type interactions between the C_6_D_6_ and the l‐**1** components, or from the reduced polarity of this solvent compared to CDCl_3_, positively influencing the hydrogen bonding energy. These effects, not mutually exclusive, were previously observed for NDI assemblies.^[^ ^37^ ^]^

To determine the dominant factor, we recorded the CD spectra of l‐**1** in (CH_3_)_2_CO, CHCl_3_ and C_6_H_6_ (Figure 2g). In cases of CHCl_3_ and C_6_H_6_ the CD spectra revealed strong positive Cotton bands centered at *λ* ≈ 380 nm, consistent with previous CD studies on helical nanotubes.^[^ ^34^ ^]^ The CD spectrum in C_6_H_6_ (Figure 2g, teal line) exhibited broadening and a bathochromic shift of ≈10 nm compared to that recorded in CHCl_3_ (Figure 2g, grey line), supporting π–π–type host:guest interactions between the nanotube and C_6_H_6_.^[^ ^45^ ^]^ An unequivocal answer about the factor responsible for increased stability of STATE 4a (hence its size), came from CD experiment in which CHCl_3_ solutions of l‐**1** (STATE 3) were titrated with two non‐polar solvents, namely C_6_H_6_ and cyclohexane (C_6_H_12_), with the latter being additionally unable to form π‐type interactions with NDI. The differences in CD intensity upon the addition of each solvent varied significantly, revealing a sharp change in the aggregation degree with 0–5% (v/v) of C_6_H_6_, contrasting with a gradual transition with up to 70% (v/v) of C_6_H_12_ (Figure S8).^[^ ^37^ ^]^

The CD studies confirm that the increased thermodynamic stability of STATE 4a is primarily driven by the template effect, supported by the reduction in solvent polarity. Specifically, C_6_H_6_ molecules fill the nanotube cavity during self‐assembly, forming a host:guest complex, with increased thermodynamic stability compared to the nanotubes observed in CHCl_3_ under the same conditions. The apparent association constant of STATE 4a is increased nearly three times, and the melting temperature *T_m_* (defined as a temperature at which *α_agg_* = 0.5) rises by 21 K.

However, the resulting complex is non‐stoichiometric, as the amount of benzene used (5% v/v up to *neat* solvent) significantly surpasses the concentration of l‐**1**, and the nanotube can easily accommodate several benzene molecules, as clearly seen in the molecular model (Figure 1b, more detailed description is available in Section 8 of the Supporting Information). This substantially complicates the direct observation of benzene binding with spectroscopic techniques. An attempt to generate STATE 4a with a trace amount of non‐deuterated C_6_H_6_ in CDCl_3_ (≈1% v/v) did show an upfield shift of the C_6_H_6_ resonance in the ^1^H NMR experiment (Figure S9a), consistent with its involvement in π‐type interactions. However, the resulting complex was found to be too dynamic to be observed on NMR‐diffusion time scale (Figure S9b). Nevertheless, the calculated interaction energy of *E_int_* = −70 kJ mol^−1^ (PBE/TZVP(D3) DFT‐ gas phase), along with the experimentally observed *ΔH*, clearly places STATE 4a below STATE 3 on the energy landscape ladder.

### State 4b–C_60_@Nanotube

The emergence of STATE 4b (Figure 1b) was triggered by the addition of a competitive guest, namely fullerene C_60_, known to interact with the surface of the NDI component (for a detailed analysis see Section 5 in Supporting Information).^[^ ^35^ ^] 1^H and ^13^C NMR analysis revealed that STATE 4b can be obtained both from STATE 3 (short nanotube) by interacting with C_60_ in CDCl_3_, and from STATE 4a upon guest exchange from C_6_D_6_ to C_60_. At high concentration (*C_NDI_* = 1.0 × 10^−2^ m), no significant differences were observed between these two methods in leading to STATE 4b. Specifically, a broadening and a downfield shift of the NDI ^1^H resonance was observed upon addition of C_60_ (Figure 2d), along with a concomitant upfield shift of the C_60_^13^C resonance by 3 p.p.m. (Figure S13). Additionally, CD spectra recorded for these mixtures revealed positive Cotton bands centered at *λ* ≈ 380 nm (Figure 2g, violet line), all results being consistent with the formation of C_60_‐filled nanotubes.^[^ ^35^ ^]^ Notably, filling of the nanotube cavity with C_60_ does not induce any major changes in the overall supramolecular structure or in the conformational changes of its subunits, as evidenced by the modeling (Figure 1b, a more detailed description is available in Section 8 of the Supporting Information). Instead, C_60_ binding results only in a further decrease in *E_int_* of the system down to −190 kJ mol^−1^ (PBE/TZVP(D3) DFT‐ gas phase), attributed to the stabilizing effect of the host:guest interactions (Figure 3b).

Further, variable temperature CD spectra recorded under dilute conditions (*C_NDI_* = 1.0 × 10^−4^ m) provided interesting mechanistic insights into the self‐assembly of STATE 4b. Surprisingly, the experiment conducted in CHCl_3_ at *C_NDI_* = 1.0 × 10^−4^ m (Figure 4c, grey trace) yielded an enthalpy release of *ΔH* = −66.7 kJ mol^−1^, and free energy release of *ΔG_298 K_* = −18.6 kJ mol^−1,^ both matching the energy levels observed for STATE 3 (an “empty” nanotube, Figure 3a), indicating the lack of formation of STATE 4b under dilute conditions, despite the presence of C_60_ in this mixture. This result suggests that C_60_ in this system acts more like a classic guest molecule, entering a pre‐formed cavity of the host, rather than a templating agent that triggers the nanotube growth. In line with this, the same reaction performed in C_6_H_6_ (Figure 4c, violet trace) yielded an enthalpy release of *ΔH* = −104.6 kJ mol^−1^, which is 17 kJ mol^−1^ lower than the energy of STATE 4a, as expected for the guest exchange reaction (STATE 4a → 4b) under thermodynamic control (Figure 1b). Notably, filling of a nanotube with C_60_ does not change its supramolecular polymerization mechanism, as indicated by the clearly sigmoidal (isodesmic) transition observed for the STATE 4b formation (Figure 4c, violet trace). Furthermore, this major decrease in enthalpy translates into only a minor change in Gibbs free energy release with *ΔG_298 K_* = −24.0 kJ mol^−1^ located barely 0.4 kJ mol^−1^ lower than that of STATE 4a. This can be reasoned by the enthalpy‐entropy compensation effect, previously observed for the helical nanotubes.^[^ ^37^ ^]^

To conclude, the formation of STATE 4b is observed only when the initial apparent *DP_N_* of a nanotube is sufficiently high. This can be achieved either by increasing the concentration or pre‐templating the assembly with C_6_H_6_.

### State 4c–C_70_@Nanotube

In contrast to the above, STATE 4c can only be observed in dilute solutions as an intermediate step in the generation of the more complex STATE 5, both triggered by the addition of the same guest, C_70_ (vide infra). The CD spectra of l‐**1** with C_70_ recorded at *C_NDI_* = 1.0 × 10^−4^ m showed a positive Cotton band centered at λ ≈ 380 nm, similar to that observed for the nanotube (Figure 2g, garnet line). Consequently, the ^1^H NMR spectrum of STATE 4c recorded at the same concentration revealed a single set of resonances, consistent with a twofold symmetry of l‐**1** (Figure 2e), indicative of the nanotube formation. This could arguably be attributed to the lack of any host‐guest interaction with C_70_ (i.e., the formation of STATE 3), especially when the ^1^H NMR analysis revealed only a minor upfield shift of 0.04 p.p.m. (albeit expected for this assembly) in comparison to STATE 3 (both at *C_NDI_* = 1.0 × 10^−4^ m, Figure S14b) and the unfeasible ^13^C NMR analysis. However, VT CD spectra revealed a substantial increase in the enthalpy release upon assembly of STATE 4c with *ΔH* = −96.2 kJ mol^−1^ and *ΔG_298 K_* = −24.0 kJ mol^−1^ in CHCl_3_ (Figure 4c, garnet trace). Notably, the transition observed for STATE 4c also follows an isodesmic pathway, and the *ΔH* places it at a similar energetic levels (Figure 3a,b) as other nanotube‐based host:guest complexes (i.e., STATEs 4a and 4b): 27 kJ mol^−1^ (in *ΔH*) / 6 kJ mol^−1^ (in *ΔG_298 K_*), compared to STATE 3 under analogous experimental conditions.

In line with this, modeling of STATE 4c revealed a decrease in *E_int_* to −220 kJ mol^−1^ (PBE/TZVP(D3) DFT – gas phase), compared to a vacant 6‐mer, without any significant structural or conformational changes – an observation reminiscent of STATE 4b.

### State 5–C_70_@Capsule

Increasing the concentration of a solution containing l‐**1** and C_70_ from *C_NDI_* = 1.0 × 10^−4^ to 1.0 × 10^−2^ m (both in CDCl_3_ and C_6_D_6_) resulted in the generation of another state, namely a C_70_‐filled hexameric capsule^[^ ^36^ ^]^ (STATE 5, Figure 1b). Namely, ^1^H NMR spectra recorded at this concentration and *T* = 298 K showed a breaking of symmetry around the NDI plane, resulting in a splitting of the NDI resonance from a singlet into four doublets (Figure 2f). Although the formation of a hexameric capsule has been observed for a similar NDI derivative,^[^ ^36^ ^]^ its detailed structure has remained largely elusive over the years, particularly due to the absence of crystallographic data and the presence of rather enigmatic NMR effects.^[^ ^36^ ^]^

**Figure 5:**
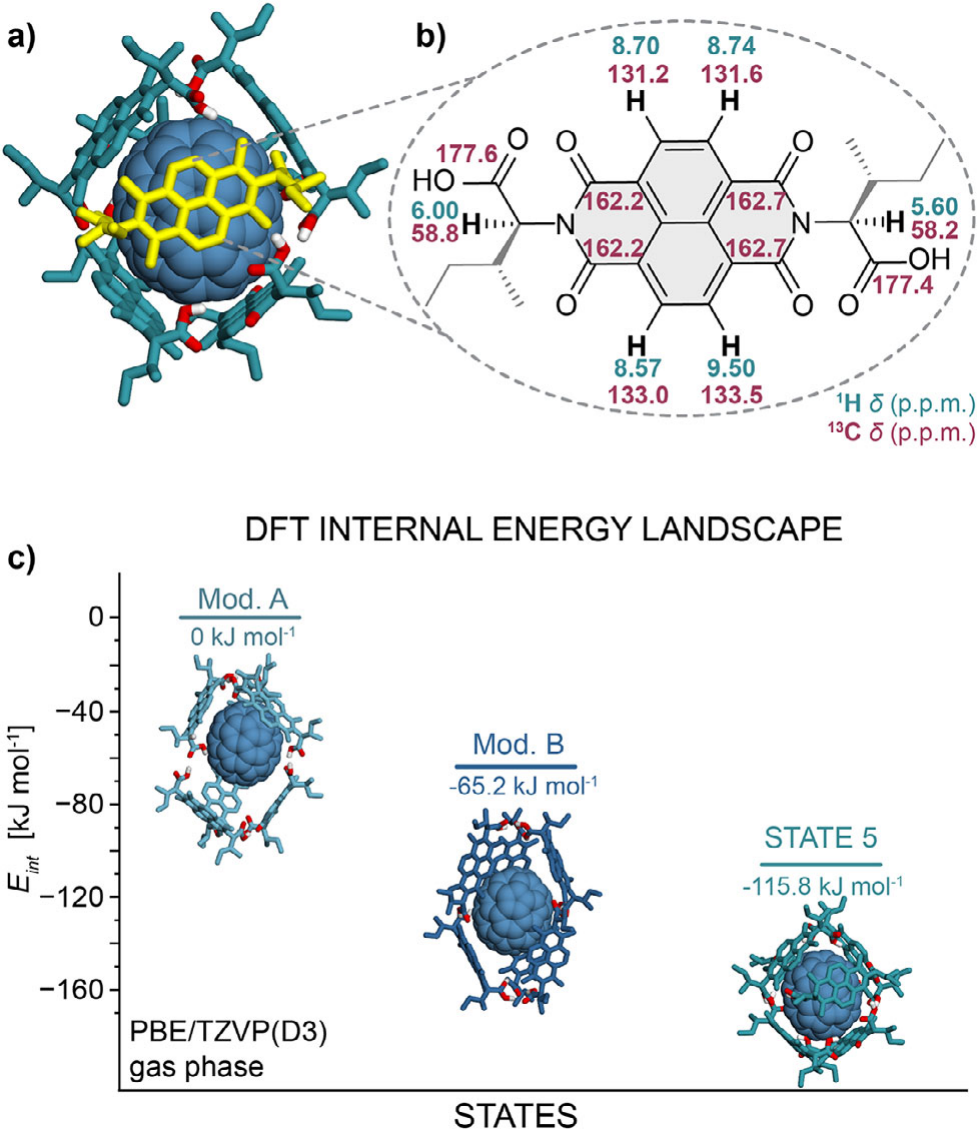
a) 3D DFT structure of STATE 5. b) Assignment of the ^1^H NMR and ^13^C NMR resonances observed for STATE 5. c) Theoretical (DFT) internal energy landscape of different STATE 5 variants.

In an effort to propose a more accurate structural model, a comprehensive 2D NMR analysis supported by the DFT molecular modeling and NMR‐GIAO calculations were performed (Details of structural model shown in Figures S20–S25). Specifically, COSY, ^1^H‐^13^C HSQC and ^1^H‐^13^C HMBC experiments enabled the assignment of individual ^1^H and ^13^C resonances around the NDI core, as shown in Figure 5b, while ^1^H‐^1^H ROESY experiment indicated dynamic exchange on the NMR time scale (for 2D NMR data see Figure S17). Notably, while the ^1^H NMR resonances of the NDI core split into two COSY pairs (4 *doublets* in total), the rest of the molecule splits into two groups located in close proximity on both the ^1^H and ^13^C scales, suggesting local symmetry disturbances rather than global changes in intermolecular interactions (vide infra).

Combinatorial molecular modelling identified three potential alternative models of STATE 5, each consistent with the observed NMR splitting pattern (Figure 2f). These alternative model structures (Mod. A, Mod. B and STATE 5) were generated by: i) superposing the planes; ii) introducing a 90° offset to one of the planes; and iii) adding a 90° offset both at the equator and along the pole axis (see Section 8 in Supporting Information for detailed analysis). Subsequently, the BSSE‐corrected PBE/TZVP(D3)‐DFT (gas phase) calculations were used to determine the internal energies *E_int_* of all configurations. The previously proposed motif (Mod. A),^[^ ^36^ ^]^ consisting of two COOH^…^(CO)HC trimers stabilized by COOH^…^HOOC bridges, was used as a baseline (Figure 5c). Surprisingly, this structure was found to be the least favorable; even its superposed variant (Mod. B) lies 65 kJ mol^−1^ lower in the energy. The most favorable structure, with *E_int_* = −115 kJ mol^−1^ (STATE 5), features a distinct, highly symmetric and compact hydrogen bonding arrangement, in which all six l‐**1** subunits are held together by COOH^…^(CO)CH interactions located at the capsule poles and vertices (Figure 5a). Despite its seemingly symmetrical structure, the capsule formed in STATE 5 is, in fact, desymmetrized due to non‐equivalent chemical environments around the NDI core (1–4 groups, Figure S18a) – an effect consistent with both the experimental NMR spectra (Figure 5b) and the NMR‐GIAO chemical shift calculations (Figure S18b).

The observed desymmetrization of an *“apparently symmetrical”* assembly is reminiscent of our previously reported hydrogen‐bonded octameric receptor for C_70_, constructed from an amino‐acid‐derived benzene‐1,3,5‐tricarboxamide scaffold.^[^ ^46^ ^]^ Noteworthy, while C_60_ and C_70_ share very similar chemical composition and binding sites, the non‐spherical and slightly elongated shape of the latter appears to favor distorted host constructs, like STATE 5. This *“shape matching”* effect is frequently observed in both supra‐^[^ ^46^, ^47^, ^48^, ^49^ ^]^ and metallo‐supramolecular systems.^[^ ^50^, ^51^ ^]^

**Figure 6:**
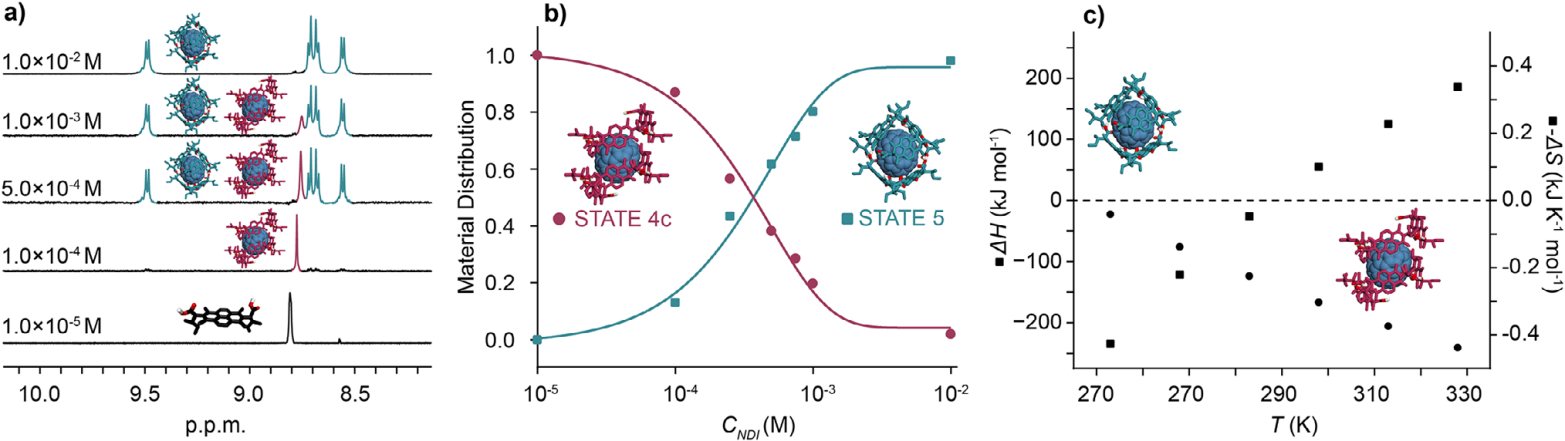
a) Part of the ^1^H NMR spectra (600 MHz, CDCl_3_, *T* = 298 K) of l‐**1** with C_70_ at different concentrations. b) Calculated dependence of a material distribution between STATEs 4c and 5 on *C_NDI_* at *T* = 298 K. c) Changes in *ΔH* and −*ΔS* against the temperature *(T)* for the formation of STATE 5.

To better understand the thermodynamics of STATE 5 formation and its transition to or from STATE 4c, as well as interconversion between the two, concentration dependent NMR experiments were carried out. Upon dilution of STATE 5 at *T* = 298 K (both in CDCl_3_ and C_6_D_6_), a gradual disappearance of the four doublets was observed, replaced by a single NDI resonance characteristic of STATE 4c, ultimately leading to the monomeric STATE 0 (Figure 6a in CDCl_3_ for data in C_6_D_6_ see Figure S15b). A plot of the integrated NDI signals as a function of *logC_NDI_* was consistent with an equilibrium between two host‐guest complexes, i.e., STATEs 4c and 5 (Figure 6b), with 50% conversion around *C_NDI_* = 5.0 × 10^−4^ m. A similar response to dilution was observed in the CD spectra, where the initially negative (at *C_NDI_* = 1.0 × 10^−2^ m, *T* = 298 K) Cotton effect, characteristic for STATE 5 (Figure 2g, turquoise line) was gradually replaced by a positive one (at *C_NDI_* = 1.0 × 10^−4^ m, *T* = 298 K), characteristic for STATE 4c (Figure 2g, garnet line). Ultimately, the CD spectrum is almost zeroed upon dilution down to *C_NDI_* = 1.0 × 10^−5^ m as a result of a full disassembly to STATE 0 (Figure S15a). DOSY NMR analysis recorded for the mixture of l‐**1** with C_70_ revealed the presence of two distinct species, i.e., C_70_‐filled nanotube (STATE 4c) with a solvodynamic radius of 0.64 nm (*D* = 6.2 × 10^−10^ m^2^ s^−1^, at *T* = 298 K) and C_70_‐filled capsule (STATE 5) with a solvodynamic radius of 0.8 nm (*D* = 5.7 × 10^−10^ m^2^ s^−1^, at *T* = 298 K) (Figures S10f,g and S11).

Based on these results, it is evident that the formation of a capsular complex (STATE 5) is not a single 6 (l‐**1**) + C_70_ ⇆ HG (host:guest) equilibrium process; instead, it involves competitive thermodynamic equilibria (STATE 4c versus STATE 5), each favored under different *C_NDI_* and *T* conditions. To further understand this complex equilibrium, VT NMR analysis was performed in CDCl_3_ at *C_NDI_* *=* 5.0 × 10^−4^ m, a concentration at which both STATEs 4c and 5 were observed at *T* = 298 K (Figure S16a). Heating of this mixture up to *T* = 328 K revealed almost full disassembly of STATE 5 and the transition into an equilibrium between STATEs 0 and 4c. Upon cooling, the capsular assembly gradually dominates the isodesmic process, as shown by the appearance of the four doublets originating from the ^1^H NMR resonances of the NDI core. The maximum conversion into STATE 5 was observed below *T* = 270 K. Based on the integrals of the individual species, the apparent *K_a_(T)* of the 6 l‐**1** + C_70_ reaction were calculated. Plotting *lnK_a_* as a function of *1/T*, using the van't Hoff relation (Figure S16b) revealed a non‐linear transition characteristic of the formation of two product assemblies with different thermodynamic characteristics, hence, *ΔH* and −*ΔS* change with temperature. Analyzing *ΔH* and −*ΔS* against *T* revealed that the process retains a negative enthalpy change throughout the transition, as expected for the hydrogen‐bonded driven assembly accompanied by π‐type host‐guest interactions (Figure 5a). However, the mysterious driving force behind the transformation of the nanotube (STATE 4c) into a capsular assembly (STATE 5) is an entropy change, which increases upon cooling, and ultimately becomes positive below *T* = 280 K (Figure 6c). The favorable entropy observed at high aggregation degrees might be explained by the critical difference between an open polymerization of a nanotube (STATE 4c), and the formation of a discrete hexameric (6 l‐**1** + C_70_) product (STATE 5). Isodesmic polymerization of STATE 4c implies that the polymer grows up to an infinite number of mers, albeit at entropic cost. This effect is negligible at low aggregation degrees (i.e., high *T* or low *C_NDI_*), since the non‐cooperative mechanism yields only short polymers with *DP_N_* = 3‐5.^[^ ^15^ ^]^ However, upon an increase in the aggregation degree, the system is forced to form longer polymeric chains, which in the case of STATE 4c, would have to also accommodate more than one guest (C_70_) molecule. After a certain threshold point is achieved, the self‐assembly of a discrete product (STATE 5) becomes entropically more favorable than the polymerization (STATE 4c), and subsequently, the thermodynamic equilibrium shifts into the formation of a C_70_‐filled hexameric capsule (STATE 5). Furthermore, because the major entropy cost is incurred during the pre‐organization stage (STATE 4c), the formation of STATE 5 is accompanied by a significant decrease in the system's free energy, reaching *ΔG_298 K_ =* −136.2 kJ mol^−1^. This substantial drop in free energy explains the preferential formation this aggregate, despite its structural complexity.

## Conclusion

In this study, we present a novel non‐covalent, multistate adaptive system that leads to the formation of several topologically distinct chiral assemblies from a single amino acid‐derived component. Our findings diverge from traditional binary‐state systems by offering a model network for exploring complex molecular behavior, thereby advancing our understanding of the adaptive and responsive characteristics of biological systems. We have demonstrated the ability to guide the self‐assembly process into five topologically distinct supramolecular assemblies across solution and solid states through the targeted application of chemical and physical triggers such as temperature, solvent, concentration, or guest molecules. These results highlight the adaptability of non‐covalent supramolecular assemblies and their advanced level of control over chirality and structural reconfiguration in response to external stimuli. Furthermore, the adaptive features of this multistate system are reflected in the diverse functionalities of the assemblies, including their capacity to accommodate different guest molecules.

From a broader perspective, the results underscore the crucial importance of meticulously selecting structurally suitable components to build complex, multistate networks of trigger‐responsive assemblies. In crafting such adaptive systems, molecules must exhibit a significant degree of flexibility, balanced with sufficient rigidity to prevent collapsing into an unstructured assembly that lacks the potential to, for example, maintain a cavity. The concept of “rigidified flexibility,” although seemingly paradoxical, is indeed optimal for developing multistate supramolecular systems. Similar to biological systems, synthetic counterparts also require a delicate balance between the stability of the generated topologies and the preservation of dynamic and responsive characteristics. Maintaining this balance is essential for achieving a competitive advantage in the enthalpy‐entropy dynamic.

## Conflict of Interests

The authors declare no conflict of interest.

## Supporting information



Supporting Information

## Data Availability

The data that support the findings of this study are available from the corresponding author upon reasonable request.
