# Supplementary material for: A Multistate Adaptive System of Topologically Distinct Chiral Assemblies
Source: Angew Chem Int Ed Engl. 2025 Jul 11;64(36):e202509903. doi: 10.1002/anie.202509903 (PMC12402909; doi:10.1002/anie.202509903)
Supplement: Supplementary file 1 — Supporting Information [file ANIE-64-e202509903-s001.docx]

*Supporting information for*

**A Multistate Adaptive System of Topologically Distinct Chiral Assemblies**

Wiktoria Adamska, Grzegorz Markiewicz, Anna Walczak, Gokay Avci, Kim E. Jelfs, Jeremy K. M. Sanders and Artur R. Stefankiewicz.

**Table of Contents**

1. Materials and Methods2

2. Synthetic procedure3

3. ^1^H and ^13^C NMR spectra of l-**1**4

4. X-ray Crystallography5

5. The supplementary characterisation of States 3-5 in solution9

6. Supplementary spectra for l-**1** aggregation11

7. Thermodynamic analysis19

8. DFT-Computational Modeling 21

9. References27

1. **Materials and Methods**

The following abbreviations were used in this file: DMSO: dimethyl sulfoxide; DMF: *N,N*-dimethylformamide; THF: tetrahydrofuran.

Chemicals were purchased from commercial suppliers, mainly Merck KGaA (Germany) and Fluorochem Ltd (UK) and used as received. NMR deuterated solvents were purchased from Deutero GmbH (Germany), at min. >99.9% isotopic enrichment (or higher) and used as received.

NMR data were performed on Bruker Co. (USA) Avance IIIHD 600 MHz spectrometers, equipped with 5 mm BBFO probe, respectively. Sample temperature was controlled with internal probe heaters and Bruker Co. (USA) BCU II variable-temperature unit. Spectra were calibrated against the residual protonated solvent signal (for ^1^H NMR DMSO-d_6_: δ = 2.50; (CD_3_)_2_CO: δ = 2.05; CDCl_3_: δ = 7.26; C_6_D_6_: δ = 7.16 for ^13^C NMR DMSO-d_6_: δ = 39.52; CDCl_3_: δ = 77.16; C_6_D_6_: δ = 128.06) and are given in p.p.m..

DOSY NMR spectra were recorded using 2D LEDbp-pulse sequence (ledbpgp2s). Δ and δ were optimised with 1D sequence (ledbpgp2s1d) to achieve sufficient signal attenuation at 95% gradient strength. Probe-specific Gradient constant was calibrated using Bruker standard sample (doped water, Bruker P/N Z10906), and cross-checked against DMSO-*d_6_* sample, in accordance with Bruker *almanac*. All DOSY experiments were recorded at *T* = 298 K. Sample temperatures were equilibrated for at least 20 min prior to the measurement to avoid temperature gradient within NMR tubes. Solvent residual peaks were used as the internal standards of diffusion rates. Diffusion coefficients were obtained from *T1/T2* analysis (Bruker TopSpin 4.0) and cross-checked with Bayesian transformation (MNova 11.0). Solvodynamic radii were calculated using Einstein-Stokes equation for spherical objects, as follows:

$$D=\frac{k_{b}T}{6\pi\eta r}$$

The following solvent viscosities were used for calculations:

*η*(CD_3_)_2_CO = 0.306 mPa.s;

*η*CDCl_3_ = 0.528 mPa.s:

*η*C_6_D_6_= 0.603 mPa.s.

To validate the solvodynamic sizes obtained across different solvents and the applicability of spherical Stokes-Einstein relations, the thus obtained radii were compared with material distributions of State 3 and 4a at *T* = 298 K and *C_NDI_* =1.0 × 10^-2^ m (Fig. S12), and were found to align with the sizes of the most abundant species averaged on the NMR-diffusion time scale.

IR spectra were obtained with Jasco Co. (Japan) 4000 FTIR spectrophotometer, operating with 4 cm^-1^ resolution, with 128 scans/spectrum, in the airtight CaF_2_ cuvette (Specac, UK) of 0.2 mm pathlength. Spectra of the pure solvents were used for subtraction.

CD spectra were recorded on Jasco Co. (Japan) *J*-1500 CD spectropolarimeter, operating at 1 nm bandwidth and 400 nm/min scanning speed. Sample temperature was controlled with 3 Peltier-type variable-temperature unit (Jasco Co., PTC-510). Measurements were performed in CHCl_3_, C_6_H_6_ or C_6_H_12_ solutions using quartz cuvettes (Hellma GMbH, Germany) with 1 mm optical path. Spectra of the pure solvents were used as the baselines.

All measurements have been performed under stagnant conditions. The temperatures were controlled within ±0.1^o^C either by the precise Peltier-type units or internal probe heater+BCU II temperature unit for NMR experiments.

1. **Synthetic procedure**

**
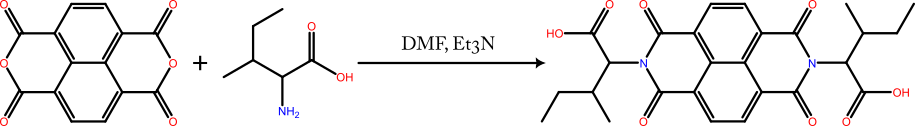
**

**Figure S1** Scheme of the synthesis of compound l-**1.**

l-**1**: l-Isoleucine (197 mg, 1.5 mmol, 2.0 equiv.) and 1,4,5,8-naphthalenetetracarboxylic dianhydride (200 mg, 0.75 mmol, 1.0 equiv.) were suspended in 6 mL of DMF in a pressure-tight 10-mL microwave vial. Then 0.5 mL of dry Et_3_N was added to the solution. Using a specialized microwave system, the reaction mixture was heated to 140 °C for 5 min, under microwave irradiation. The solution was then added while being stirred to 200 mL of 1 m HCl. Using a Buchner funnel, the resultant suspension was filtered after coagulating for two hours. The solid was then dried in a vacuum after being rinsed with 100 mL of deionized water. The product was obtained as a light-brown solid in 84% yield. ^1^H NMR (600 MHz, DMSO-d_6_) δ (ppm): 12.82 (s, 2H), 8.76 (s, 4H), 5.23-5.21 (d, 2H), 2.48-2.44 (m, 2H), 1.32-1.24 (m, 2H), 1.20-1.19 (d, 6H), 0.95-0.86 (m, 2H), 0.77-0.73 (t, 6H); ^13^C NMR (600 MHz, DMSO-d_6_) δ (ppm): 170.16, 162.50, 131.52, 128.46, 125.78, 57.93, 33.08, 24.70, 17.95, 10.89.

1. **^1^H and ^13^C NMR spectra of l-1**


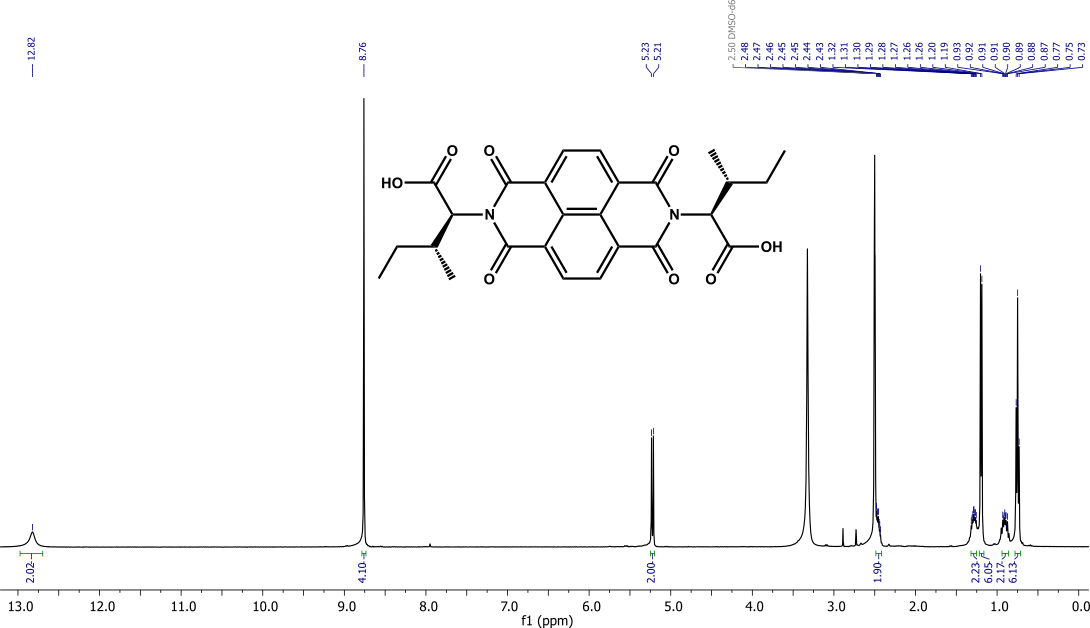


**Figure S2** ^1^H NMR (600 MHz, 298 K, 1.0 × 10^-2^ m, DMSO-d_6_) spectrum of l-**1**.


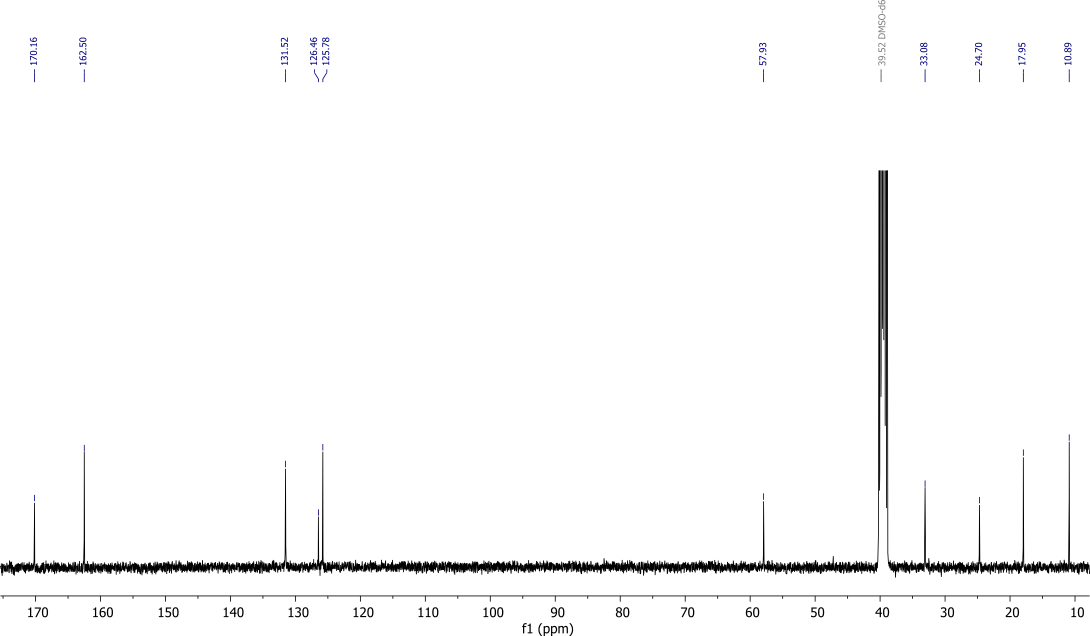


**Figure S3** The ^13^C NMR spectra (600 MHz, 298 K, 1.0 × 10^-2^ m, DMSO-d_6_) of l-**1**.

1. **X-ray Crystallography**

**POLYMORPH 1**

The crystal structure of **Polymorph 1** has already been described in the book chapter: N. Ponnuswamy, A. R. Stefankiewicz, J. K. M. Sanders, G. D. Pantoş, *Top. Curr. Chem*. **2012**, 322, 217-260. However, the CIF of the structure was not deposited in the CCDC database at that time. We currently do not have access to source files, making it impossible to correct the structure and include structure factor data. In the current work, we compare the structure of **Polymorph 1** to **Polymorph 2**, which is why we decided to deposit the structure in the CCDC database in this form.

Based on the information contained in the CIF file: the structural studies for **Polymorph 1** was performed using an a Nonius Kappa CCD. Xray data were collected at 150(2)K using graphite-monochromated MoKα radiation (λ = 0.71073 Å) with the thin slice ω- and φ-scan technique. Data collection was performed using Collect^[S1]^, and cell refinement was conducted using HKL Scalepack^[S2]^. The structure was solved with SIR-92^[S3]^ and refined by full-matrix least-squares against F2 with the SHELXL refinement package^[S4]^ based on Least Squares minimization. The -COOH hydrogen atoms were not located but placed in chemically reasonable positions (one disordered over two possible sites). Two of the three dichloromethane solvate molecules are poorly resolved, each disordered over at least two sites - these moieties were modelled with extensive use of bond length restraints and common, isotropic displacement parameters.

**POLYMORPH 2**

The structural studies for **Polymorph 2** was performed using an a New Xcalibur EosS2 diffractometer equipped with a CCD (Charge-coupled Device) detector. Xray data were collected at 293(2)K using graphite-monochromated MoKα radiation (λ = 0.71073 Å) with the ω-scan technique. Data reduction, UB-matrix determination, and absorption correction were performed using the CrysAlisPro software.^[S5]^ Using Olex2,^[S6]^ the structure was solved by direct methods with ShelXT^[S7]^ and refined by full-matrix least-squares against F2 with the SHELXL refinement package^[S4]^ based on Least Squares minimization. Non-hydrogen atoms were refined anisotropically, while the H-atoms were located in idealized positions by molecular geometry and refined as riding groups with Uiso(H) = 1.2 Ueq (for C-carriers) and Uiso(H) = 1.5 Ueq (for O-carriers). For one water molecule, it was impossible to reliably determine the positions of hydrogen atoms, so they were not taken into account in the final model.

Selected structural parameters are reported in Table S1. The data have been deposited in the Cambridge Crystallographic Data Collection (CCDC) with deposition numbers CCDC 2358687 and 2358692. These data can be obtained free of charge via www.ccdc.cam.ac.uk/data_request/cif, by emailing data_request@ccdc.cam.ac.uk, or by contacting The Cambridge Crystallographic Data Centre, 12 Union Road, Cambridge CB2.

**Polymorph 2 Alert level A:**

PLAT417_ALERT_2_A Short Inter D-H..H-D H5A..H5A. 1.67 Ang.

RESPONSE: This alert is related to the hydrogen atoms of lattice water molecules. Geometric constraints (e.g., eadp) have been imposed on the oxygen atoms in water molecules. Additionally, it was difficult to locate hydrogen atoms accurately using X-ray data because crystals did not diffract past approximately 1.02 Å resolution.

**Polymorph 2 Alert level B:**

[PLAT084_ALERT_3_B](javascript:makeHelpWindow(%22PLAT084.html%22)) High wR2 Value (i.e. > 0.25) ................... 0.39 Report

[PLAT340_ALERT_3_B](javascript:makeHelpWindow(%22PLAT340.html%22)) Low Bond Precision on C-C Bonds ............... 0.01462 Ang.

RESPONSE: High value of wR2 indicator and low bond precision were caused by poor quality of the crystal - crystal did not diffract past approximately 1.02 Å resolution.

[PLAT417_ALERT_2_B](javascript:makeHelpWindow(%22PLAT417.html%22)) Short Inter D-H..H-D H6A..H6B. 2.08 Ang. y,-x,z = 4_555 Check

[PLAT420_ALERT_2_B](javascript:makeHelpWindow(%22PLAT420.html%22)) D-H Bond Without Acceptor O6--H6B. Please Check

RESPONSE: This alert is related to the hydrogen atoms of lattice water molecules. It was difficult to locate hydrogen atoms accurately using X-ray data because crystals did not diffract past approximately 1.02 Å resolution.

[PLAT097_ALERT_2_B](javascript:makeHelpWindow(%22PLAT097.html%22)) Large Reported Max. (Positive) Residual Density 0.98 eA-3

[PLAT601_ALERT_2_B](javascript:makeHelpWindow(%22PLAT601.html%22)) Unit Cell Contains Solvent Accessible VOIDS of 144 Ang**3

RESPONSE: The highest peak in the final difference map is just 0.98 e/A3 and no model for any solvent could be found.

**Table S1** Crystal data and structure refinement for Polymorph 1 and Polymorph 2.

|  | 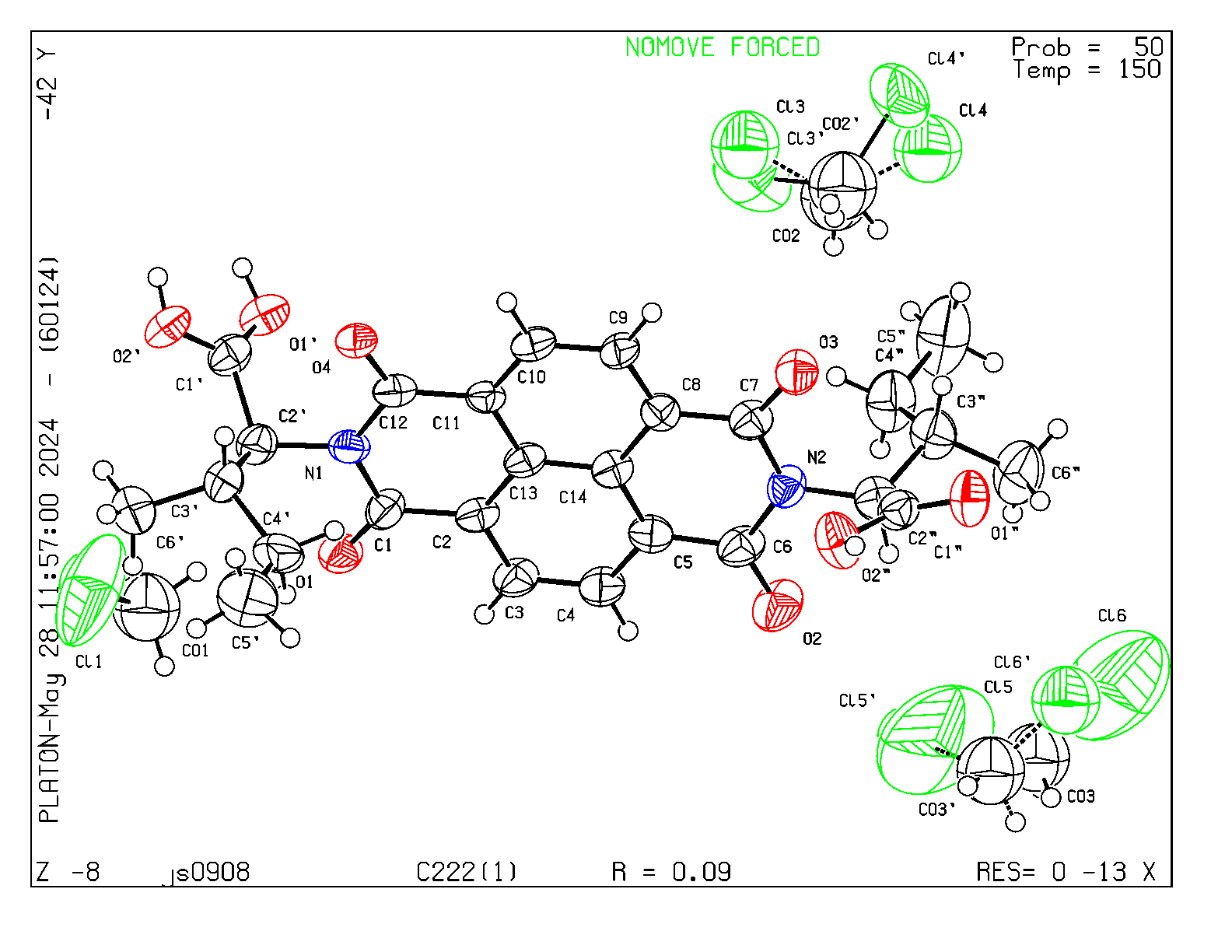 | 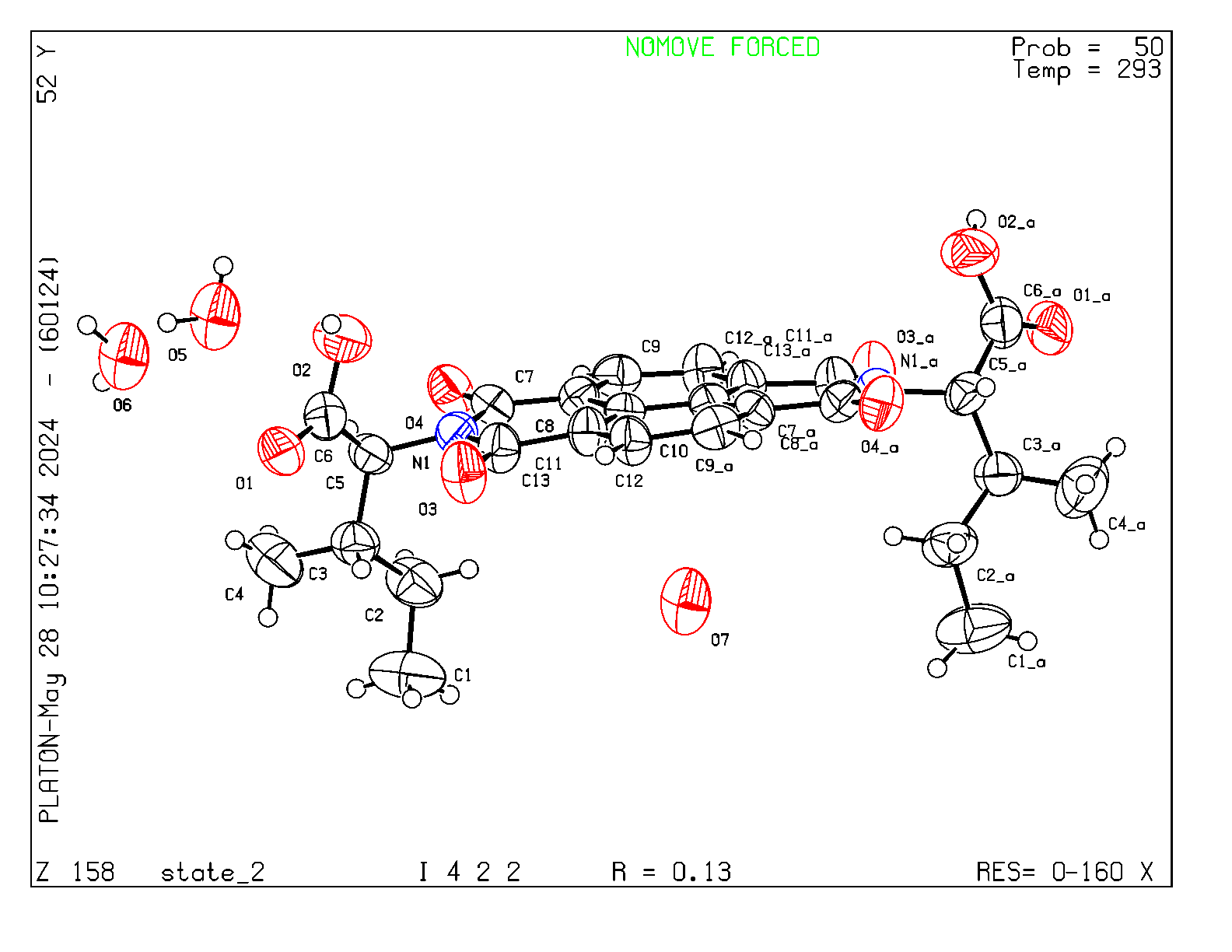 |
| --- | --- | --- |
| Identification code | **Polymorph 1** | **Polymorph 2** |
| CCDC number | 2358692 | 2358687 |
| Empirical formula | C_29_H_32_Cl_6_N_2_O_8_ | C_104_H_136_N_8_O_49_ |
| Formula weight | 749.27 | 2282.20 |
| Temperature/K | 150(2) | 293(2) |
| Crystal system | orthorhombic | tetragonal |
| Space group | C222_1_ | I422 |
| a/Å | 10.7851(2) | 14.6712(4) |
| b/Å | 14.2592(3) | 14.6712(4) |
| c/Å | 46.119(2) | 30.1896(12) |
| α/° | 90.00 | 90.00 |
| β/° | 90.00 | 90.00 |
| γ/° | 90.00 | 90.00 |
| Volume/Å^3^ | 7092.5(4) | 6498.1(4) |
| Z | 8 | 2 |
| ρ_calc_g/cm^3^ | 1.403 | 1.166 |
| μ/mm^‑1^ | 0.532 | 0.093 |
| F(000) | 3088.0 | 2416.0 |
| Crystal size/mm^3^ | 0.28 × 0.21 × 0.14 | 0.02 × 0.02 × 0.005 |
| Radiation | MoKα (λ = 0.71073) | MoKα (λ = 0.71073) |
| 2Θ range for data collection/° | 7.1 to 44.84 | 6.354 to 50.042 |
| Index ranges | -11 ≤ h ≤ 11, -13 ≤ k ≤ 15, -48 ≤ l ≤ 49 | -17 ≤ h ≤ 17, -17 ≤ k ≤ 17, -35 ≤ l ≤ 35 |
| Reflections collected | 10691 | 75999 |
| Independent reflections | 4102 [R_int_ = 0.0446, R_sigma_ = 0.0461] | 2859 [R_int_ = 0.1697, R_sigma_ = 0.0646] |
| Data/restraints/parameters | 4102/30/412 | 2859/0/188 |
| Goodness-of-fit on F^2^ | 1.024 | 1.407 |
| Final R indexes [I>=2σ (I)] | R_1_ = 0.0857, wR_2_ = 0.2238 | R_1_ = 0.1342, wR_2_ = 0.3627 |
| Final R indexes [all data] | R_1_ = 0.1036, wR_2_ = 0.2404 | R_1_ = 0.1860, wR_2_ = 0.3920 |
| Largest diff. peak/hole / e Å^-3^ | 0.82/-0.67 | 0.98/-0.46 |
| Flack parameter | -0.1(2) | -0.5(10) |

The crystallization of l-**1** from CH_2_Cl_2_, a molecule exhibiting weak hydrogen-bonding properties, yields a compound of the composition (l-**1**)_3_×CH_2_Cl_2_. The resulting structure (Fig. S4) prominently features "classical" hydrogen bonding interactions between carboxylic acid groups, exerting a significant influence. Reciprocal OH^...^O interactions between carboxylic acid groups are responsible for the development of a right-handed helical, monoperiodic polymer along the (001) direction. The helix does not have solvent occupancy even if solvent molecules, particularly CH_2_Cl_2_, are present in the crystalline lattice. This is probably because of the helix's small diameter (~11 Å) and the hydrogen bonding network's tight packing. It is also noteworthy that there are no stacking interactions between the NDI units. The nearest parallel NDI planes stay apart by a significant amount, around 7 Å, and do not overlap when projected. It's interesting to note that Ile alkyl groups partially interpose between these planes, adding to the structural intricacy.

Understanding the precise roles of solvent molecules in the crystal structure is challenging, primarily due to factors such as the lack of proton localization for water molecules and the disorder of certain dichloromethane molecules. However, potential interactions, such as dichloromethane-CH^...^O (carboxyl) interactions and "halogen bonds" involving dichloromethane-Cl and carboxyl-O (O2"), warrant further investigation.


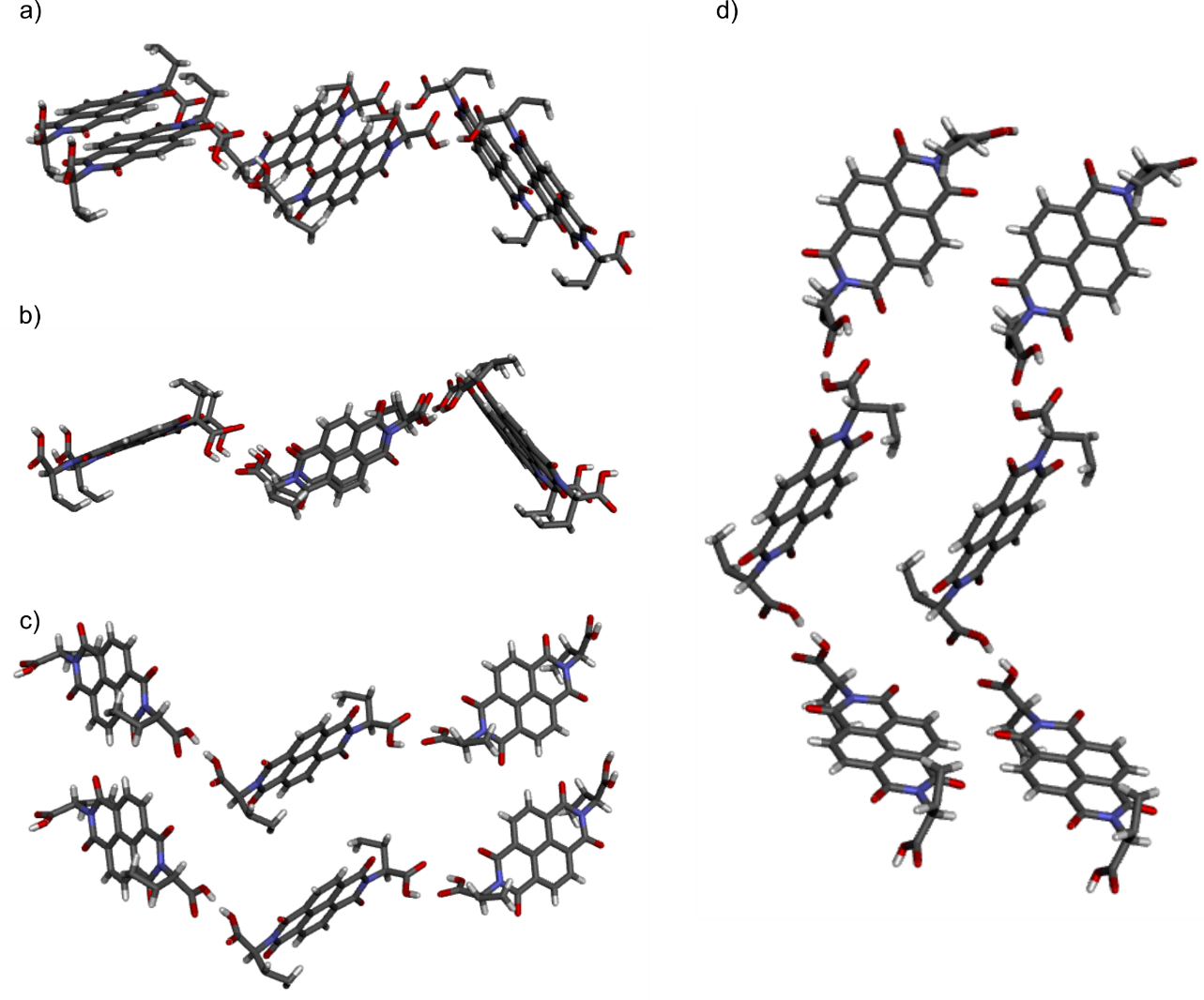


**Figure S4** Views of a helical polymer formed by carboxylic acid groups in the structure of (l-**1**)_3_×CH_2_Cl_2_.

In an attempt to crystallize l-**1** from tetrachloroethane, a solvent known not to disrupt solute self-hydrogen-bonding interactions, prolonged solvent evaporation under standard atmospheric conditions led to the isolation of crystals identified as the simple hydrate, (l-**1**)_4_×H_2_O. Subsequent X-ray structure determination of one such crystal revealed a markedly distinct structure compared to the dichloromethane solvate (Fig. S5). This second structure features l-**1** units adopting their *syn* conformation, with two inequivalent yet very similar conformations observed. The crystal exhibits a layered arrangement, with thick layers parallel to [010]. These layers consist of diperiodic polymers formed by interactions between carboxyl groups, while sheets of water molecules separate them. Notably, the hydrogen bonding involving the carboxylic acid groups assumes a completely different configuration. Quartets of these groups, derived from two pairs of the inequivalent l-**1** units, form cyclic arrays through *syn-anti* linking.


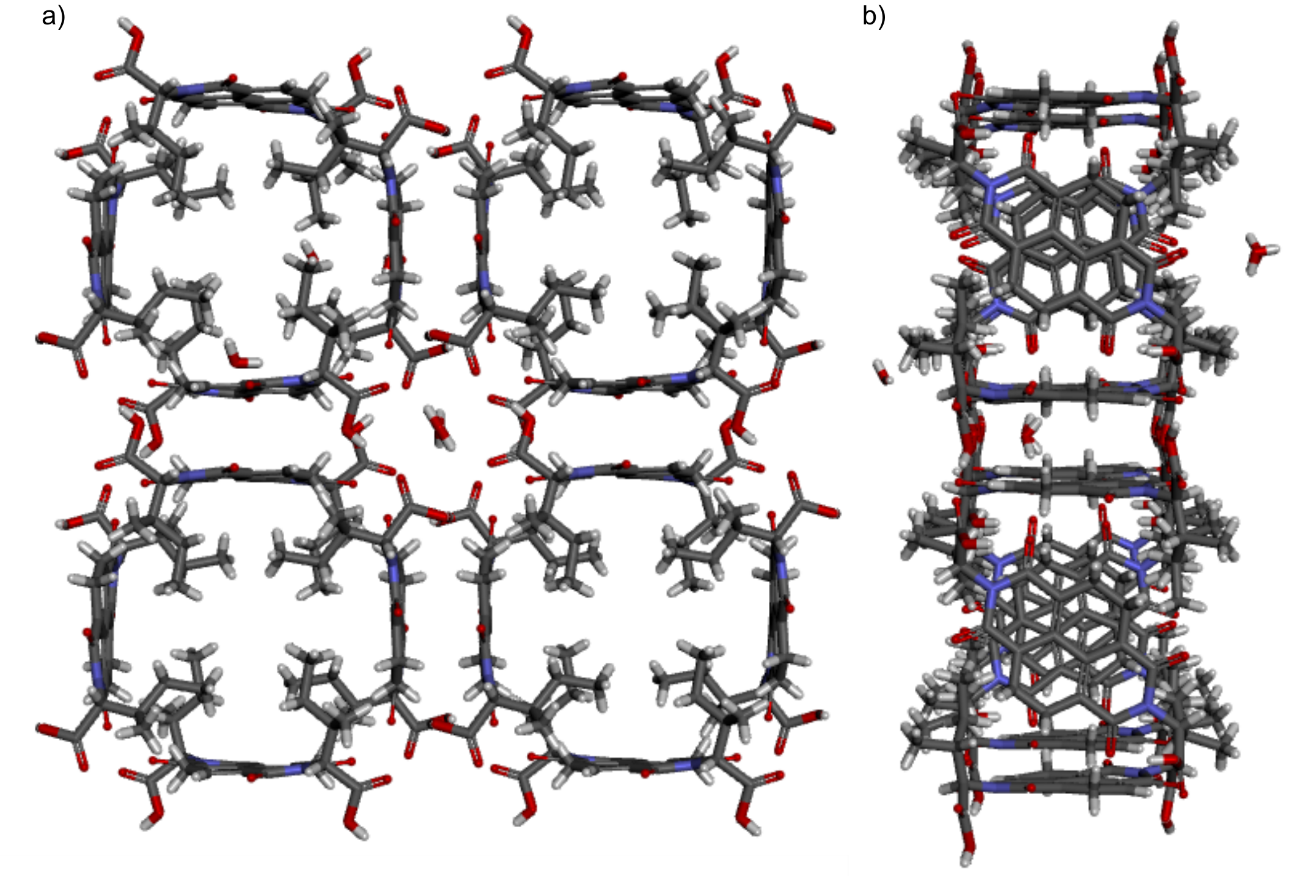


**Figure S5** Partial view of the structure of (l-**1**)_4_×H_2_O, showing its layered nature.

1. **The supplementary** **characterisation of States 3-5 in solution**

**State 3:** The ^1^H NMR spectrum (Fig. 2a and S6a) of l-**1** in CDCl_3_ recorded at 298 K and *C_NDI_* = 1.0 × 10^−2^ m revealed a single set of resonances, consistent with twofold molecular symmetry. Compared to the spectrum recorded in a highly polar solvent (i.e. (CD_3_)_2_CO), the NDI proton of l-**1** shifts upfield by 0.1 p.p.m. (to 8.70 p.p.m.), while the α-CH signal shifts downfield by the same margin (to 5.45 p.p.m.). ^13^C NMR shows a downfield shift of ~5 p.p.m. for the COOH resonance (Fig. S6b), indicating the formation of COOH^...^HOOC bridges. Moreover, Circular Dichroism (CD) spectra revealed a positive Cotton band centred at λ ≈ 380 nm (Fig. 2g, grey line) and both the Variable Temperature CD analysis as well as Concentration Dependent ^1^H NMR confirmed that the self-assembly proceeds *via* isodesmic supramolecular polymerisation mechanism.

The results are in line with those observed in solution for the analogous NDI derivatives,^[S8, S10, S12]^ whose self-assembly in CDCl_3_ gives an isodesmic growth of helical nanotubes, thus unambiguously confirmed the formation of this type of assembly also by l-**1** in CDCl_3._

To further support this, the FT-IR analysis and DOSY NMR spectra were performed. In line with the expected structure, FT-IR spectra revealed a bathochromic shift of the *ν*C=O band from 1750 to 1725 cm^−1^ (Fig. S7a), while DOSY-NMR (Fig. S10) confirmed that all resonances originate from single species, with a solvodynamic radius of 0.76 nm in CDCl_3_ (nanotube, State 3) and 0.43 nm in (CD_3_)_2_CO (monomer, State 0).

**STATE 4a:** The ^1^H NMR spectrum recorded at 298 K and *C_NDI_* = 1.0 × 10^−2^ m (Fig. 2c, S6 in SI) exhibited a significant upfield shift of approximately 0.6 p.p.m. for the NDI proton, suggesting the involvement of both π-π stacking and CH^...^O hydrogen bonding in the molecular structure. Additionally, a downfield shift of about 6 p.p.m. for the COOH resonance in ^13^C NMR supports this observation (Fig. S6). Furthermore, Circular Dichroism (CD) spectra revealed a positive Cotton band centred at λ ≈ 380 nm (Fig. 2g, teal line) and both the Variable Temperature CD analysis as well as Concentration Dependent ^1^H NMR confirmed that the self-assembly proceeds *via* isodesmic supramolecular polymerisation mechanism.

The results are in line with those observed in solution for the analogous NDI derivatives,^[S8, S10, S12]^ whose self-assembly gives an isodesmic growth of the guest-filled helical nanotubes, thus unambiguously confirmed the formation of this type of assembly also by l-**1** in C_6_D_6._

Furthermore, FT-IR analysis revealed a bathochromic shift in the *ν*C=O band from 1750 to 1725 cm^−1^ (Fig. S7a), further indicating the presence of hydrogen bonding interactions. DOSY NMR confirmed an increase in the solvodynamic radius of State 4a to 0.94 nm, in comparison to State 3 (Fig. S10c).

**STATE 4b:** The ^1^H NMR spectrum recorded at 298 K and *C_NDI_* = 1.0 × 10^−2^ m exhibited a broadening and downfield shift of the NDI proton upon the addition of C_60_ (Fig. 2d), accompanied by an upfield shift of the C_60_ ^13^C resonance by approximately 3 p.p.m. (Fig. S13). Additionally, the CD spectra of these mixtures revealed positive Cotton bands centered at λ ≈ 380 nm (Fig. 2g, violet line), indicating the formation of C_60_-filled nanotubes. Moreover, the Variable Temperature CD analysis confirmed that the self-assembly proceeds *via* isodesmic supramolecular polymerisation mechanism.

The results are in line with those observed in solution for the analogous NDI derivatives,^[S8, S10, S12]^ whose self-assembly gives an isodesmic growth of the guest-filled helical nanotubes, thus unambiguously confirmed the formation of this type of assembly also by l-**1** with C_60_ in CDCl_3._

**STATE 4c:** The CD spectra of l-**1** with C_70_ at a concentration of *C_NDI_* = 1.0 × 10^−4^ m exhibited a positive Cotton band centered at λ ≈ 380 nm, similar to that observed for the nanotube (Fig. 2g, garnet line). The ^1^H NMR spectrum of State 4c, recorded at the same concentration, revealed a single set of resonances, indicating the twofold symmetry of l-**1** (Fig. 2e) and further confirming nanotube formation. Furthermore, the Variable Temperature CD analysis confirmed that the self-assembly proceeds *via* isodesmic supramolecular polymerisation mechanism.

The results are in line with those observed in solution for the analogous NDI derivatives,^[S8, S10, S12]^ whose self-assembly gives an isodesmic growth of the guest-filled helical nanotubes, thus unambiguously confirmed the formation of this type of assembly also by l-**1** with C_70_ in CDCl_3._

**STATE 5:** The ^1^H NMR spectrum of l-**1** with C_70_ at a concentration of *C_NDI_* = 1.0 × 10^−2^ m revealed a loss of symmetry around the NDI plane, as evidenced by the splitting of the NDI resonance from a singlet into four doublets (Fig. 2f). This was accompanied by an upfield shift of the C_70_ ^13^C resonance by approximately 2.5 p.p.m. (Fig. S13). Additionally, the CD spectrum of State 5 displayed a negative and broaden Cotton effect at λ ≈ 380 nm (Fig. 2g, turquoise line).

These results are reminiscent to those observed in solution for an analogous NDI derivatives with C_70_, ^[S9, S10]^ *i.e*. templated formation of a hexameric receptor for C_70_ fullerene, thus unambiguously confirmed the formation of this type of assembly also by l-**1** with C_70_ in CDCl_3._

Furthermore, DOSY NMR analysis of the l-**1** and C_70_ mixture confirmed the presence of two distinct species: a C_70_-filled nanotube (State 4c) with a solvodynamic radius of 0.64 nm (D = 6.2 × 10^−10^ m^2^ s^−1^ at 298 K), and a C_70_-filled capsule (State 5) with a solvodynamic radius of 0.74 nm (D = 5.7 × 10^−10^ m^2^ s^−1^ at 298 K) (Fig. S11a, b).

1. **Supplemental spectra for l-1 aggregation**


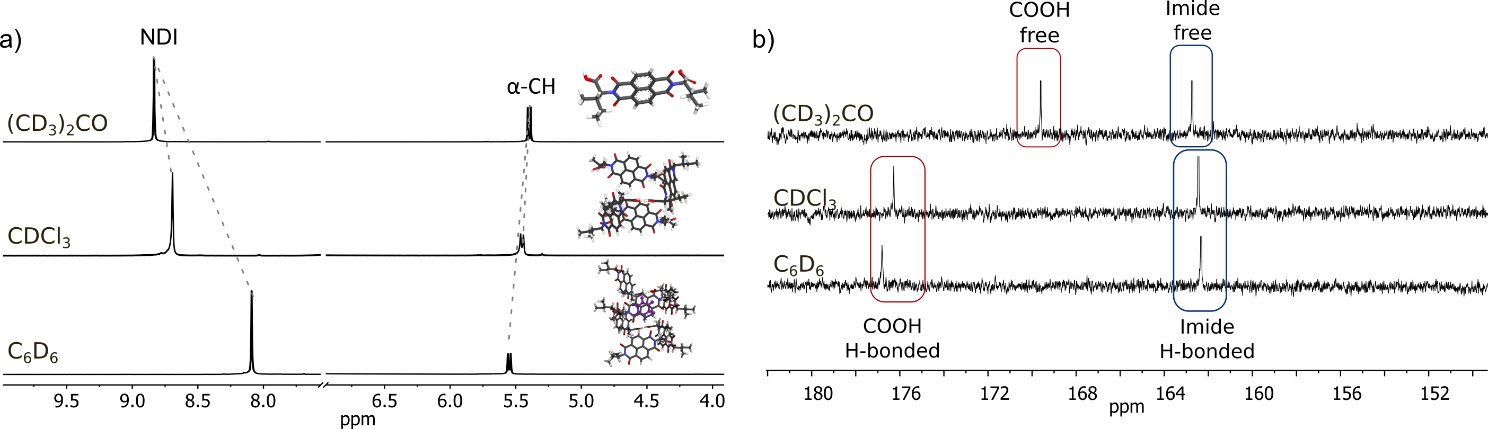


**Figure S6** a) Part of the ^1^H NMR spectra (600 MHz, 298 K, 1.0 × 10^-2^ m) of l-**1** in different solvents; b) Part of the ^13^C NMR spectra (600 MHz, 298 K, 1.0 × 10^-2^ m) of l-**1** in different solvents.


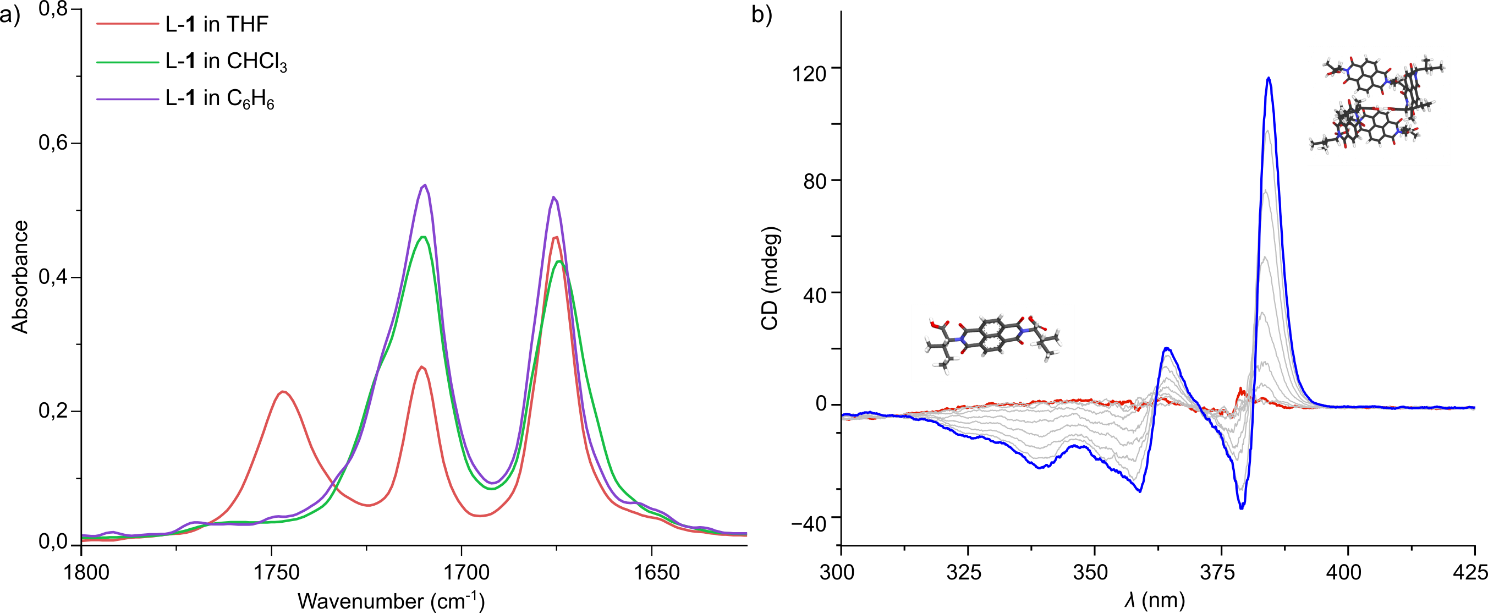


**Figure S7** a) FT-IR (298 K, 1.0 × 10^-2^ m) spectrum of l-**1** in THF, CHCl_3_ and C_6_H_6_ b) The temperature dependency CD spectra of L-**1** in (CDCl_3_).


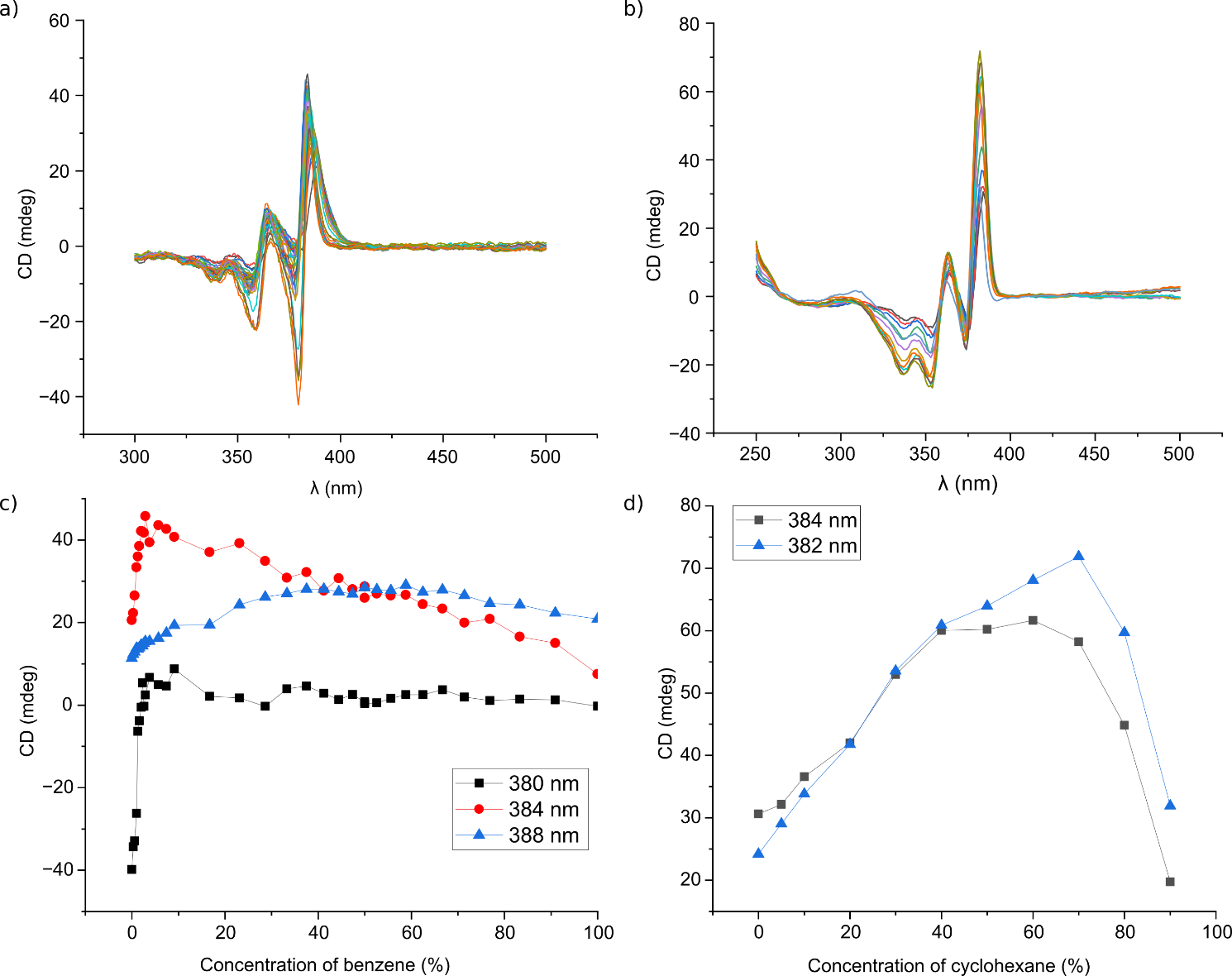


**Figure S8** The CD spectra of l-**1** in CHCl_3_ with addition of a) C_6_H_6_ b) C_6_H_12_ c) changes in the CD intensity upon addition of C_6_H_6_ d) changes in the CD intensity upon addition of C_6_H_12_.


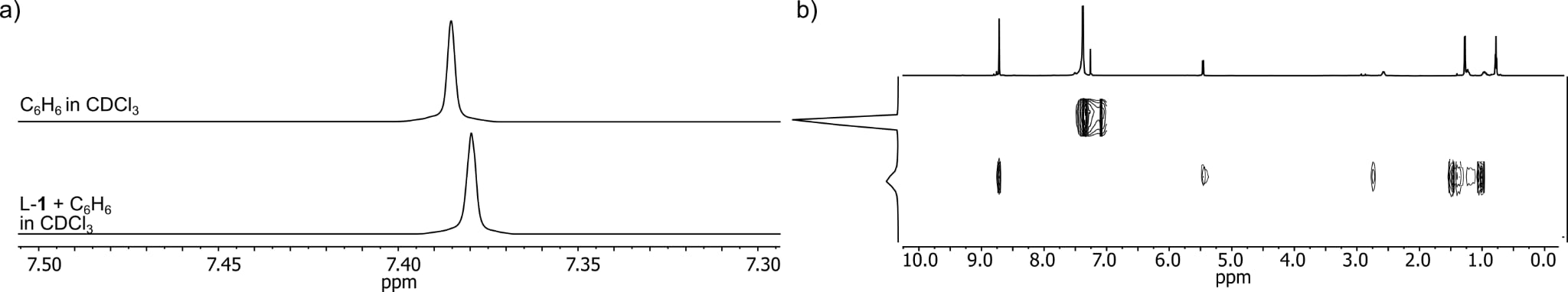


**Figure S9** a) ^1^H NMR resonance of C_6_H_6_ in CDCl_3_ (1% v/v) without (top) and with the presence of l-**1**; at *C_NDI_* =1.0 × 10^-2^ m (bottom). b) DOSY NMR spectra of l-**1** with C_6_H_6_ in CDCl_3_ (1% v/v).


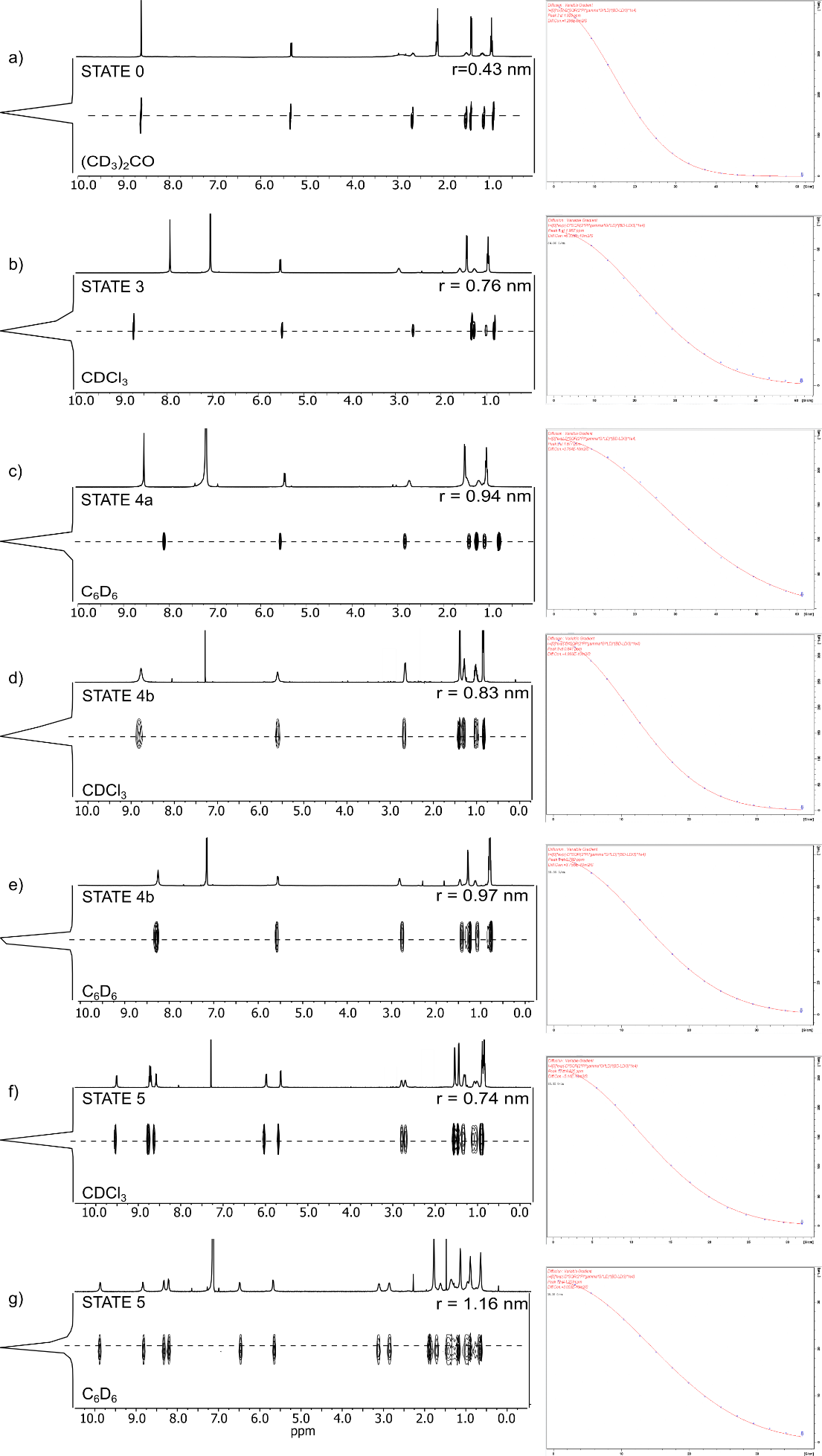


**Figure S10** DOSY NMR (600 MHz, 298 K, *C_NDI_* =1.0 × 10^-2^ m) spectra of l-**1** corresponding to: a) STATE 0; b) STATE 3; c) STATE 4a; d) STATE 4b in CDCl_3_; e) STATE 4b in C_6_D_6_; f) STATE 5 in CDCl_3_; g) STATE 5 in C_6_D_6_**.**


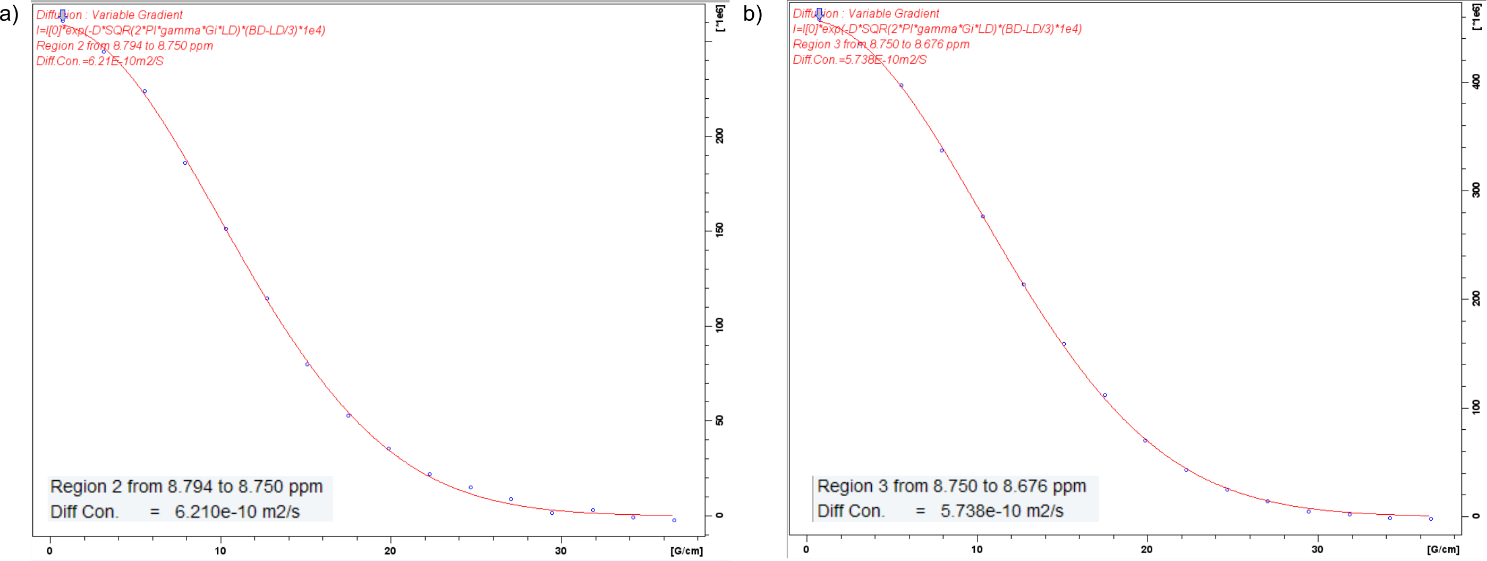


**Figure S11** The peak-separated fitting curves obtained from DOSY NMR (600 MHz, 298 K) spectrum of STATE 4c:STATE 5 mixture. a) STATE 4c peak; b) STATE 5 peak.


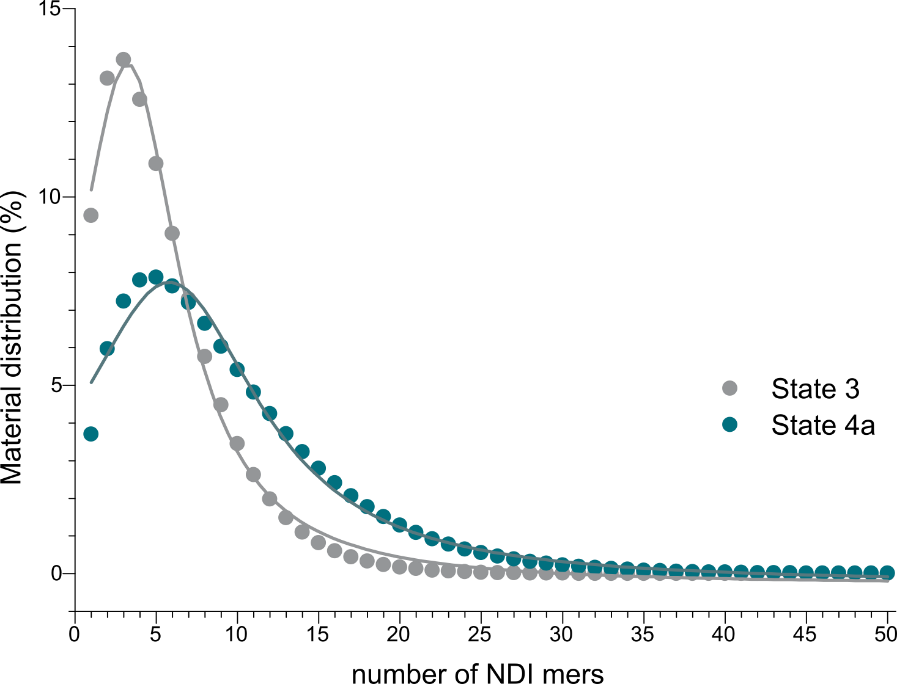


**Figure S12** Plot of material distribution (%) as a function of the number of l-**1** mers for
State 3 (grey *K = 3000 m^-1^*) and State 4a (teal, *K = 8000 m^-1^*) calculated for the species at *T* = 298 K and *C_NDI_* =1.0 × 10^-2^ m.


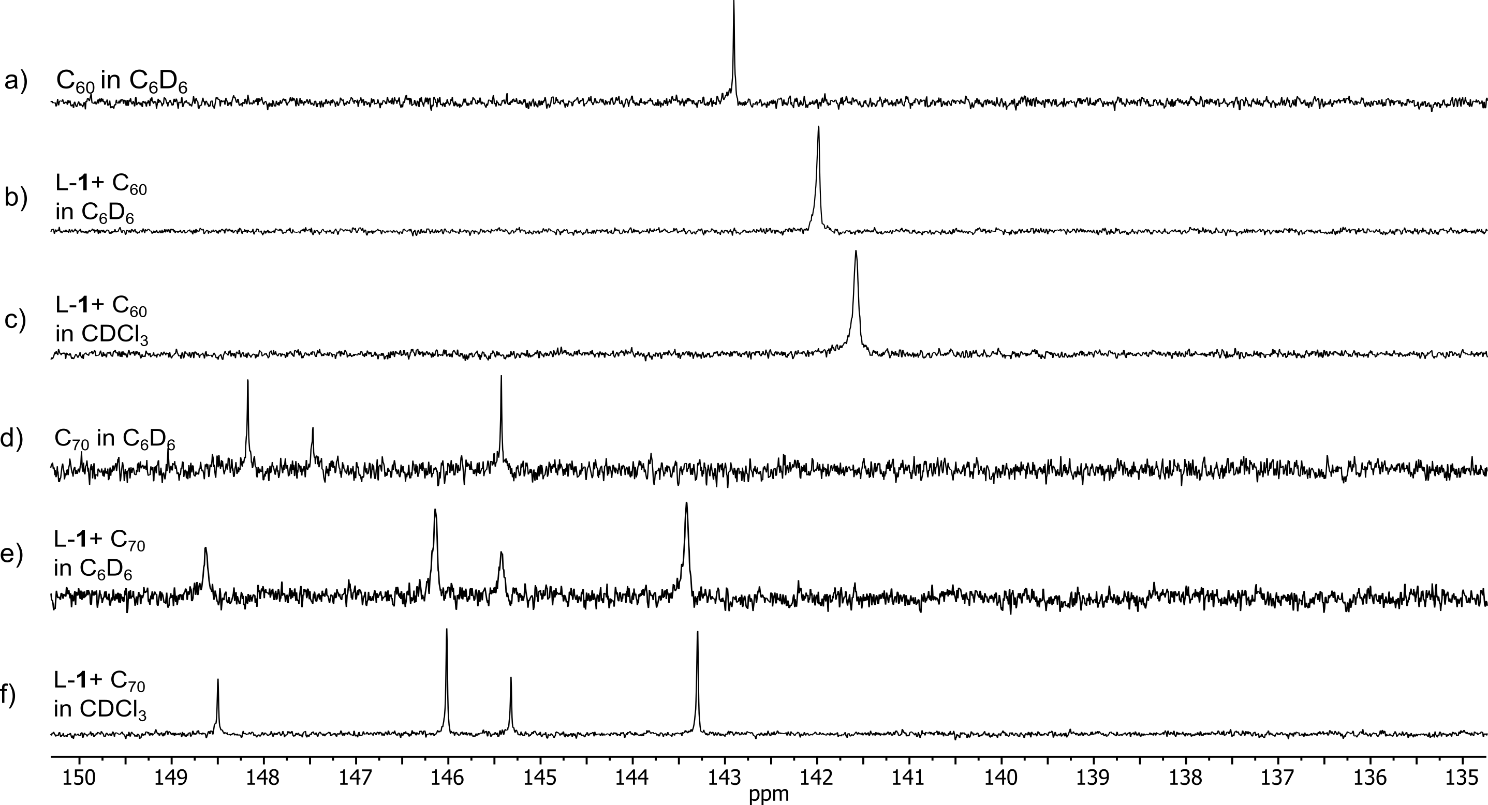


**Figure S13** Part of the ^13^C NMR spectra (600 MHz) of a) fullerene C_60_ in C_6_D_6_ b) l-**1** with C_60_ in C_6_D_6_ c) l-**1** with C_70_ in CDCl_3_ d) fullerene C_70_ in C_6_D_6_ e) l-**1** with C_70_ in C_6_D_6_ f) l-**1** with C_70_ in CDCl_3_.

**
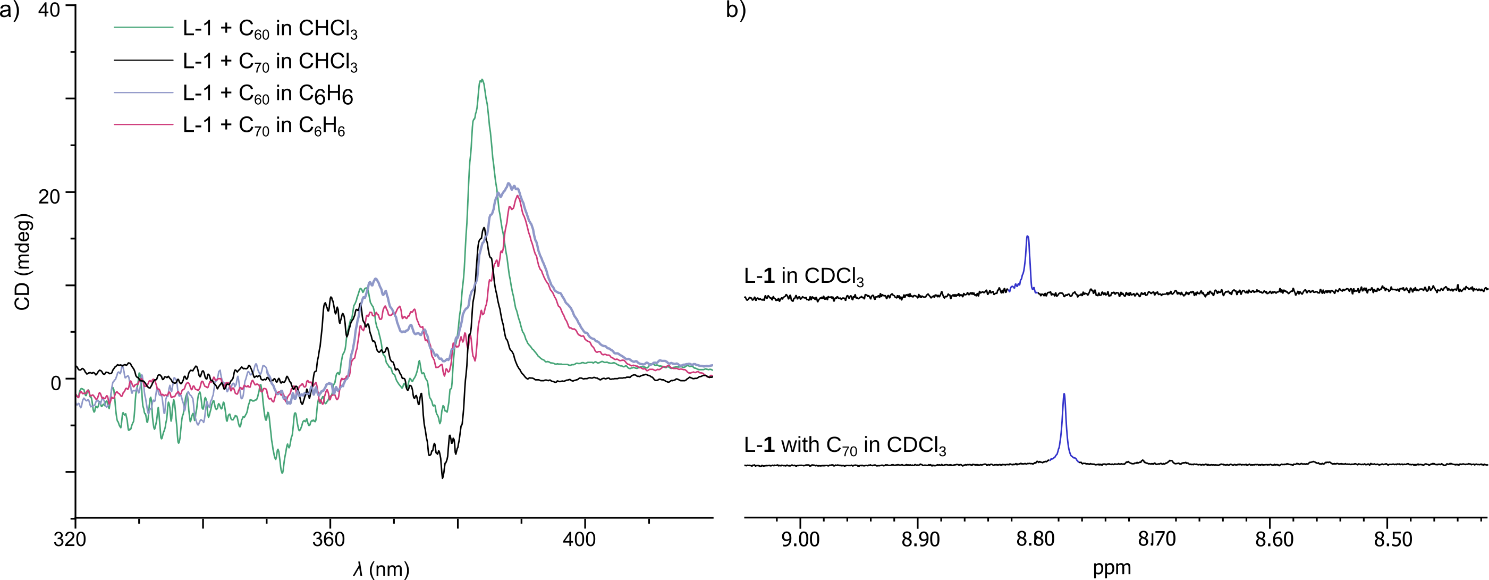
Figure S14** a) CD spectra (298 K, *C_NDI_* = 1.0 × 10^-4^ m) of l-**1** with C_70_ and C_60_ in CHCl_3_ and C_6_H_6_; b) ^1^H NMR spectra of l-**1** at *C_NDI_* = 1.0 × 10^-4^ m.


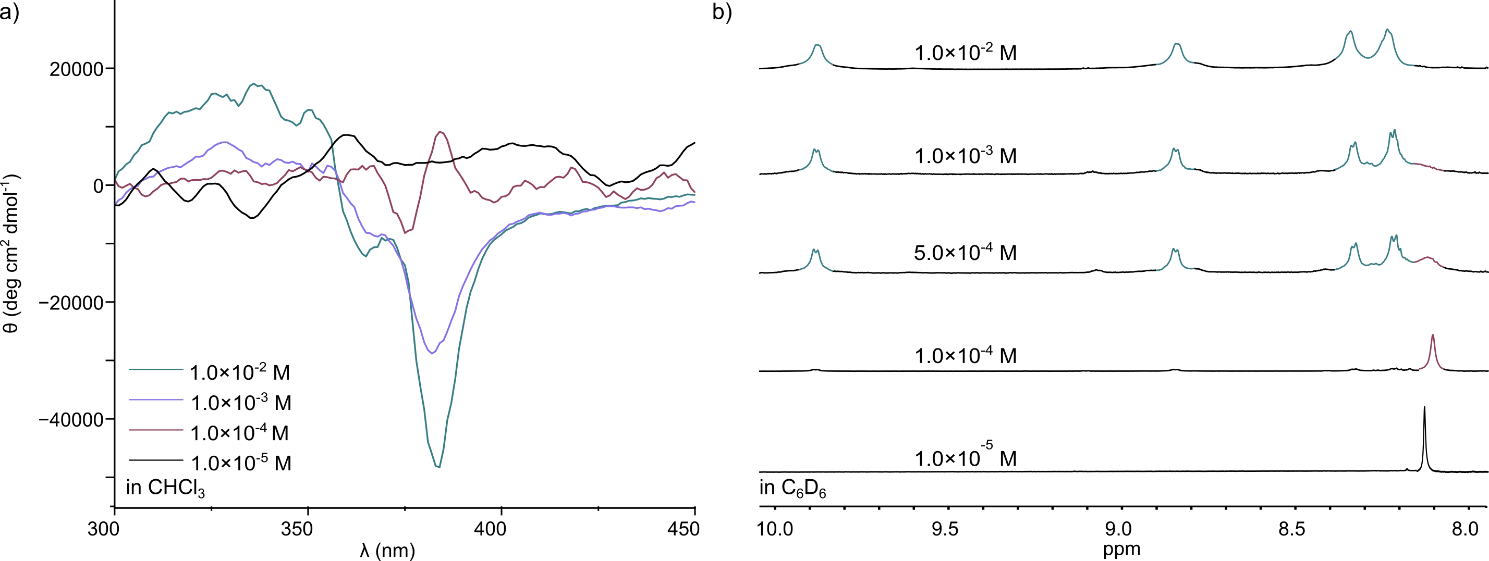


**Figure S15** a) CD spectra (298 K, *C_NDI_* = 1.0 × 10^-4^ m) of l-**1** with fullerene C_70_ in CHCl_3_ at different concentration at 298 K, b) ^1^H NMR spectra (600 MHz, 298 K) of l-**1** with C_70_ fullerene in C_6_D_6_ at different concentrations : STATE 4c (garnet), STATE 5 (turquoise), STATE 0 (black).


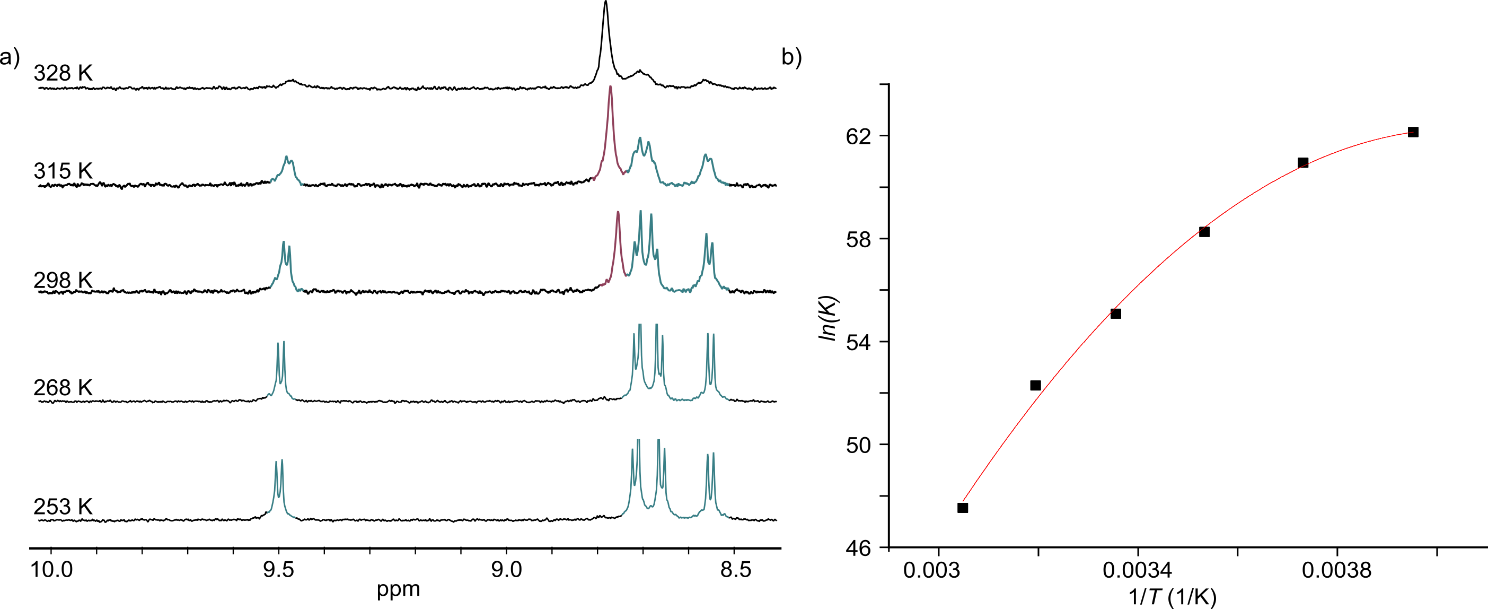


**Figure S16** a) Variable temperature ^1^H NMR spectra (600 MHz, *C_NDI_* = 5.0 × 10^-4^ m) of l-**1** with C_70_ fullerene in CDCl_3_: STATE 4c (garnet), STATE 5 (turquoise), STATE 0 (black); b) The van’t Hoff plot for the formation of STATE 5


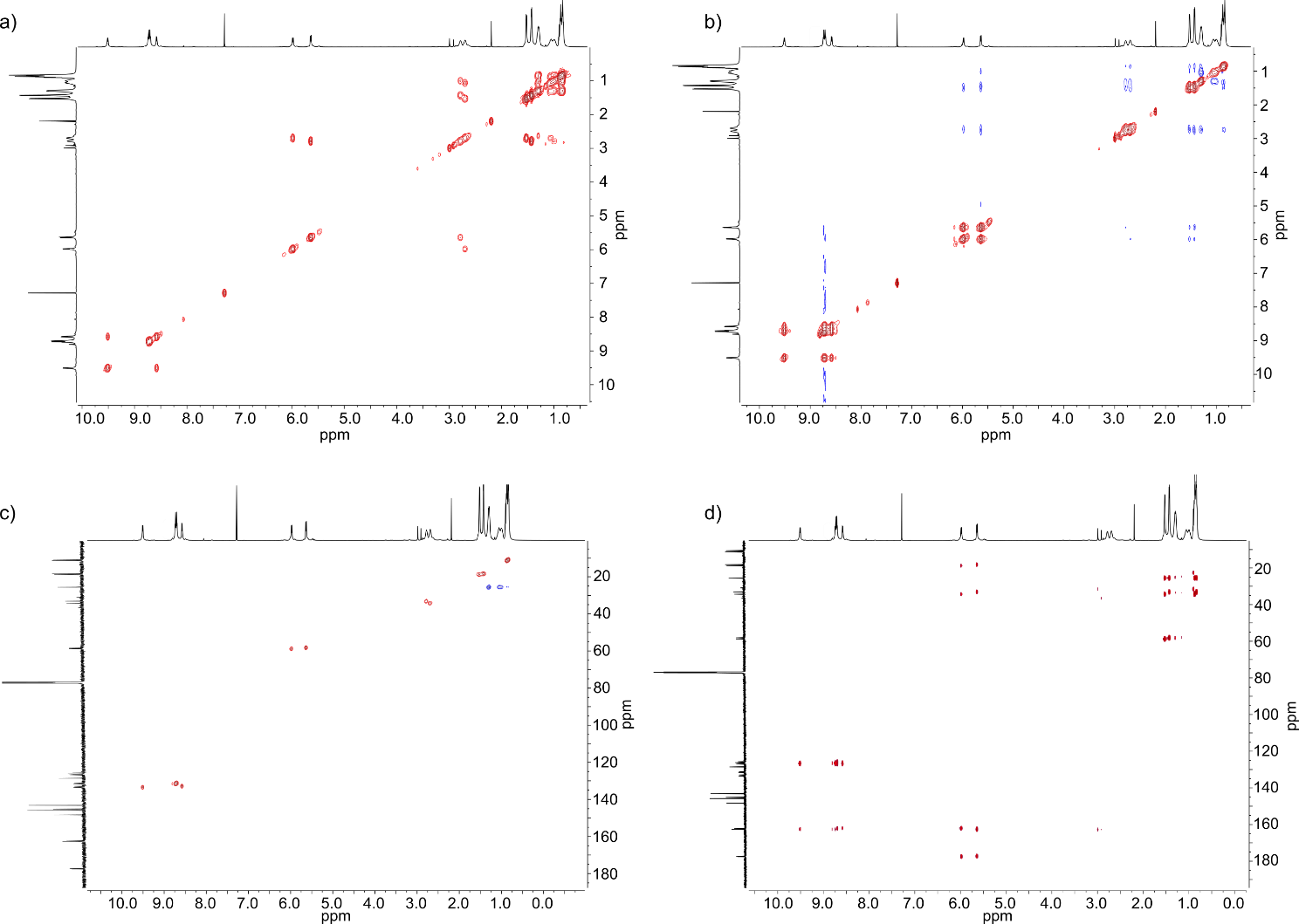


**Figure S17** a) COSY NMR spectrum (600 MHz, *C_NDI_* =1.0 × 10^-2^ m) of l-**1** with C_70_ in CDCl_3_; b) ROESY NMR spectrum (600 MHz, *C_NDI_* =1.0 × 10^-2^ m) of l-**1** with C_70_ in CDCl_3_; c) HSQC NMR spectrum (600 MHz, *C_NDI_* =1.0 × 10^-2^ m) of l-**1** with C_70_ in CDCl_3_; d) HMBC NMR spectrum (600 MHz, *C_NDI_* =1.0 × 10^-2^ m) of l-**1** with C_70_ in CDCl_3._


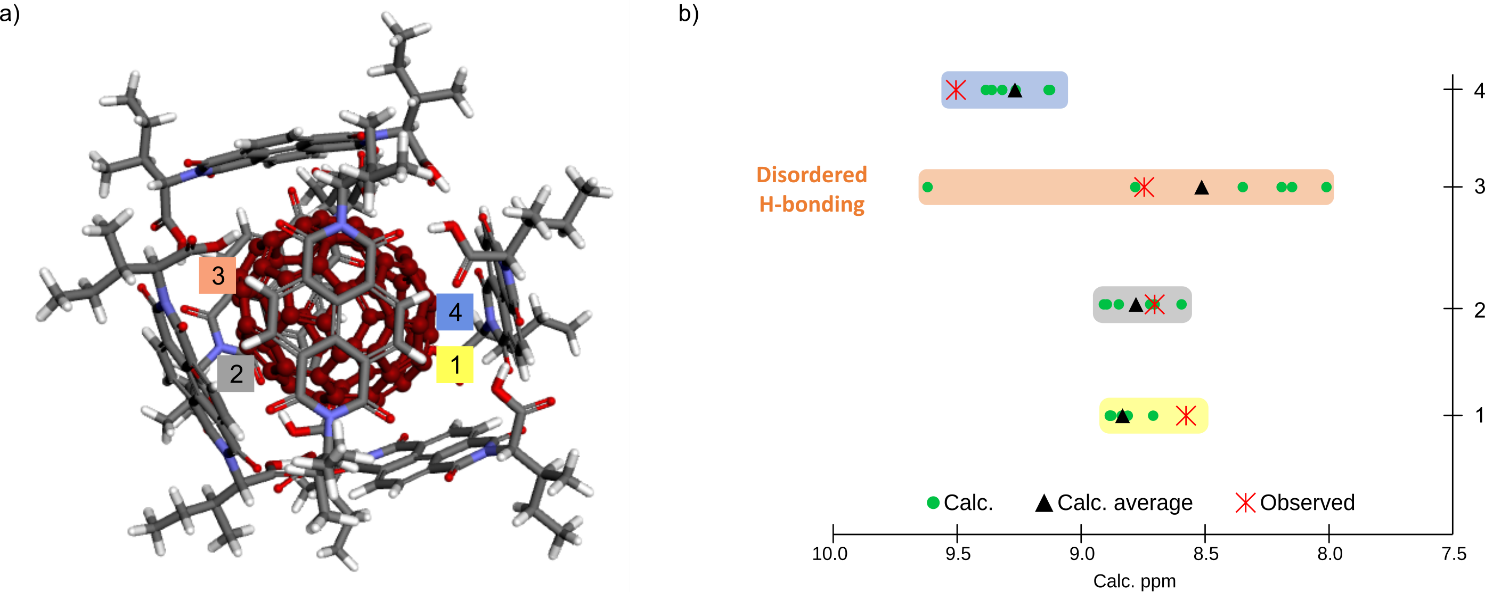


**Figure S18** 3D DFT structure of STATE 5 with assigned NDI hydrogens and symmetry splitting. b) Calculated
^1^H NMR shifts of NDI resonances (NMR GIAO) along with the experimentally observed values.

1. **Thermodynamic analysis**

Concentration-dependent NMR analysis of STATEs 3 and 4a

Chemical shift of the NDI resonance was plotted as a function of log *C_NDI_* (Fig. 3a). After normalisation (as Δ δ p.p.m.) data were fitted to the C-dependent isodesmic polymerisation model,^[S11]^ which has been previously applied for NDI-based helical nanotubes.^[S12]^

Namely, *C-*dependent data were fitted to the following equation:

$$\alpha\left( T \right)=1-\frac{2KC_{T}+1-\sqrt{4KC_{T}+1}}{2K^{2}{C_{T}}^{2}}$$

Where:

*α* = degree of aggregation (0.0 ≤ *α* ≤ 1.0)

*K* = association constant

*C_T_* = total concentration of the sample

All the fits were performed using *‘non-linear curve fit’* function in Origin Pro 8.0, and thus *K* values were obtained.

Variable Temperature CD analysis of STATEs 3, 4a, 4b, 4c

All spectra were replotted and rescaled from Jasco Spectra Manager files using OriginPro 8.0. After normalization, (as *α*) VT CD data were fitted to the isodesmic model,^[S13]^ which has been previously applied for NDI-based helical nanotubes.^[S12, S14]^

Namely, *T-*dependent data were fitted to the following equation:

$$\alpha\left( T \right)\cong\frac{1}{1+\exp(-0.908 \Delta H_{m} \frac{T-T_{m}}{R{T_{m}}^{2}})}$$

Where:

*α* = degree of aggregation (0.0 ≤ *α* ≤ 1.0)

*T* = sample temperature

*T_m_ =* melting temperature (*T* at α = 0.5)

*ΔH_m_ =* melting enthalpy

*R* = gas constant

All the fits were performed using *‘non-linear curve fit’* function in Origin Pro 8.0 and thus *ΔH_m_* and *T_m_* values were obtained.

From *α(T)*, the degree of polymerization *DP_N_ (T)* can be calculated directly, via:

$${DP}_{N}(T)=\frac{1}{\sqrt{1-\alpha\left( T \right)}}$$

From the thus obtained *DP_N_ (T), the association constant K can be calculated, via:*

$${DP}_{N}\left( T \right)=\frac{1}{2}+\frac{1}{2}\sqrt{4KC_{T}+1}$$

Subsequently, the Gibbs free energy *ΔG* and entropy *ΔS* can be calculated directly, via:

$\Delta G=-RT lnK$ $\Delta G=\Delta H-T\Delta S$

Concentration dependent and variable temperature NMR analysis of STATE 5

NMR spectra were recorded on a Bruker Avance III HD 600 MHz spectrometer equipped with external BCU II temperature controller. Standard ^1^H NMR pulse sequence (zg) has been used, with Lorentzian line broadening *lb* = 0.5 Hz. Spectra were integrated manually in Mnova 11.0 software. Material distributions were calculated based on initial concentrations of regents (*C_NDI_* and *C_C70_*) and ^1^H NMR integrals.

The self-assembly of STATE 5 can be expressed as follows:

6 (l-**1**) + C_70_ ⇆ (STATE 5)

And the equilibrium constant *K* of this reaction as:

$$K=\frac{[STATE 5]}{\left[ L\boldsymbol{1} \right]^{6}[C_{70}]}$$

To verify that the system is indeed under thermodynamic control as well as to validate the above established protocol, the system was first subjected to a C-dependent NMR analysis at 298 K. The stock solution of STATE 5 in CDCl_3_, with an initial l-**1** concentration of *C_NDI_* = 1.0 × 10^-2^ m, was prepared and subsequently diluted down to *C_NDI_* = 1.0 × 10^-5^ m. As shown in Fig. S19, the calculated concentrations of individual species under equilibrium show a classical Boltzman-type material distribution and the thus obtained association constants *K* fall within a same range (3–9 × 10^22^ m^-6^).


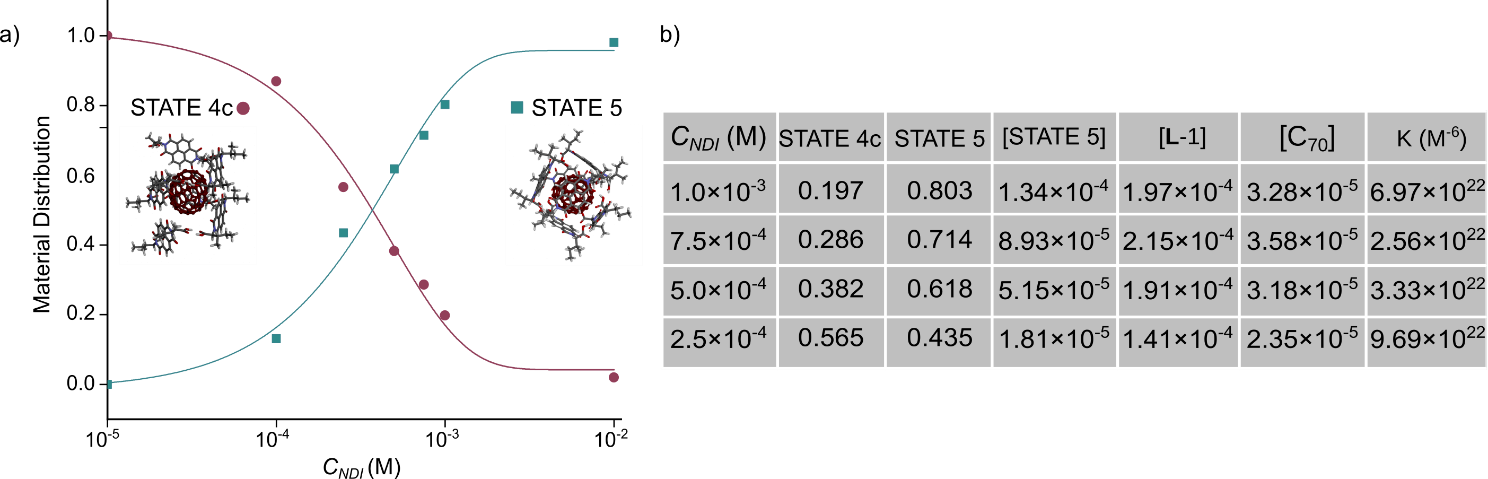


**Figure S19** a) Calculated dependence of a material distribution between STATEs 4c and 5 on *C_NDI_* at 298 K. b) Determination of association constants at different concentration.

For variable temperature NMR analysis a solution at *C_NDI_* = 5.0 × 10^-4^ m. The association constants *K*(T) at particular temperatures *T* were calculated using the protocol described above. Thermodynamic parameters for the self-assembly of a STATE 5 were obtained by plotting *lnK* as a function of 1/*T*. The thus obtained van’t Hoff plot has shown a clear deviation from the linear trend, as expected for the formation of two products under thermodynamic equilibrium.^[S15]^

A first-order approximation of the van’t Hoff relation was applied and the data were fitted to the quadratic van’t Hoff equation, as follows: ^[S15]^

$$lnK=\alpha+ \frac{\beta}{T}+\frac{\gamma}{T^{2}}$$

Where:

$\Delta H= -R \left( \beta+\frac{2\gamma}{T} \right)$ and $\Delta S= R \left( \alpha+\frac{\gamma}{T^{2}} \right)$

1. **Computational Modeling**

**Methods:**

The hydrogen bonded capsules and monomers and n-mers, l-**1**_8_, (the L-isoleucine derivative of 1,4,5,8-naphthalene-tetracarboxylic) were constructed with the Supramolecular Toolkit (*stk*) software.^[S16]^ To build the monomers and the 6-mers, the monomers were adapted from a previously reported structure by introducing L-isoleucine groups to the 1 and 5 positions of the 6-mer structure.^[S8]^ Hydrogen bonded polymer networks (State 3, State 4a, State 4b and State 4c) were modelled as 6-mers, as such structures sufficiently encapsulated all guest molecules. State 5 was modeled as a supramolecular capsule, composed of 6 monomer units of l-**1**_8_. From here onwards, each state will be collectively referred to as “supramolecular assemblies” for clarity. The guest molecules, benzene, C_60_ and C_70_ were placed at the centroid of each supramolecular assembly, which were geometry optimized with the CP2K plane-wave density functional theory (DFT) package. For each of the sampled starting configurations, the structures were then geometry optimised to find the nearest local minimum. Density Functional Theory (DFT) calculations with the CP2K software package^[S19]^ were employed using the PBE functional,^[S20]^ def2-TZVP basis set,^[S21]^ a grid size of 400 Ry and a relative cut-off value of 100 Ry. Non-bonded interactions were included using the Grimme-D3 dispersion correction.^[S22]^ The L-BFGS (Limited memory Broyden-Fletcher-Goldfarb-Sanno) algorithm^[S23]^ was used for geometry optimisation with convergence criteria of a maximum step size of 0.003 Å, root mean squared step size of 0.0015 Å, maximum gradient of 0.0004 Hartree/Bohr and a root mean squared of gradient as 0.003 Hartree/Bohr. A basis set superposition error correction was applied for host-guest cases as implemented within CP2K, to correct any basis function overlaps, with the following equation:

$E_{BSSE}={(E}_{A}(AB)-E_{A}(A))+ {(E}_{A}(AB)-{(E}_{B}(B)$)

Where $E_{A}(A)$ and $E_{B}(B)$ correspond to isolated host and guest states, and $E_{A}(AB)$ corresponds to host-guest state.

The formation energy for 6-mer capsule formation was calculated with the following formula:

$$E_{formation}=6 x E_{Monomer}-E_{Six-mer}$$

Where $E_{formation}$ corresponds to formation energy of a hydrogen bonded polymer network, $E_{Monomer}$, corresponds to the single point energy of the l-**1**_8_ monomer, and $E_{Six-mer}$ corresponds to the single point energy of the 6-mer structures. The binding energy for guests were calculated with the following formula:

$$E_{binding}=E_{Complex}-E_{Host}- E_{Guest}$$

Where $E_{binding}$ corresponds to the binding energy, $E_{Host}$ corresponds to the single point energy of the host, $E_{Guest}$ corresponds to the single point energy of the guest (C_60_, C_70_ or C_6_H_6_), and $E_{Complex}$ corresponds to the host-guest complex energy for supramolecular assemblies, which where retrieved from single points energies from the conformations, reoptimized as a complex.

We developed a computational approach to identify the lowest energy gas phase conformation of each supramolecular capsule, which would match to the experimentally observed capsules. It is experimentally challenging to obtain crystal structures for hydrogen-bonded supramolecular capsules due to dynamic behaviour, which is investigated computationally with our approach. To build State 5 supramolecular capsules, three out of six monomers were placed at the center of facets of tetrahedral polyhedra, leaving one face of the tetrahedra empty, which was used as a base for the equator zone. The remaining three monomers were replicated with a C_2_ mirror symmetry operation, which completes the supramolecular capsule. The two tetrahedra were joined by aligning base faces of two tetrahedral polyhedra in Macromodel^[S17]^ software, therefore forming a 6-mer capsule. Each monomer was rotated sequentially from 0° to 120° with a step size of 5° about the vector between the centroid of the polyhedral and the monomer to enumerate all potential capsule configurations using the ChimeraX software.^[S18]^ The supramolecular capsule construction steps and monomer rotation steps are illustrated in Fig S20. For each given capsule, this rotation method ensured a fine grid scan that would find configurations of the capsule that maximize the number of hydrogen bonds between the molecular components, with a total of 46 656 configurations considered.


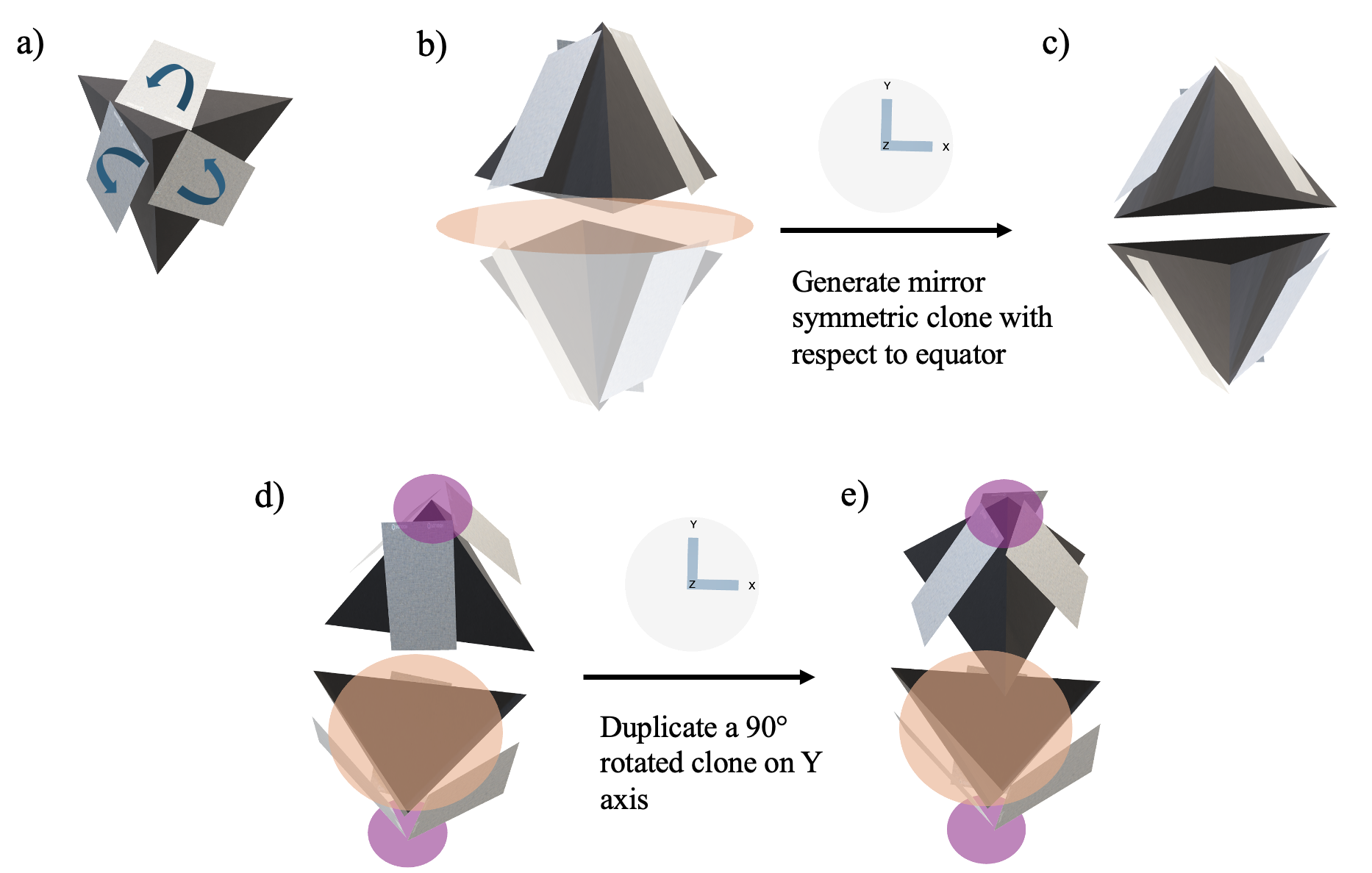


**Figure S20** An example of the computational setup for modelling the arrangements of monomers in a supramolecular capsule formation for l-**1**_8_. The perylene based monomers are simplified as plates. a) using the simplified representation, placing the monomers around the platonic solid (tetrahedra) facets, where the plates are rotated through the axis parallel to each corresponding tetrahedra face, b) representation of 6-mer construction step, where the assembly constructed in step a) is cloned with respect to the equator plane mirror symmetry (shown in orange), c) Resultant supramolecular capsule, d) Illustration of offset compared to the equator e) Resultant offset supramolecular capsule. The poles are shown with purple discs for clarity.

Alternative models (Mod. A, Mod. B and STATE 5) were devised by i) adding a 90 degree offset to one of the planes ii) superposing the planes and iii) adding a 90 degree offset both to the equator and the pole axis (Figure S21). Supramolecular assemblies are illustrated in Fig S24 and Fig S25 with hydrogen bonded networks highlighted.


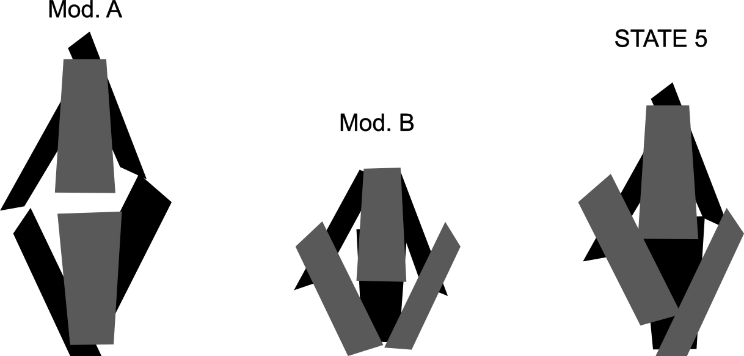


**Figure S21** Presentation of molecular distribution models of l-**1** in case of State 5. The rectangles correspond to abstraction of l-**1**_8_ monomer placements, the coloring hue between gray (front) and black (back) are to assist with depth perception.

**NMR calculations:**

NMR chemical shifts were calculated at the DFT level for State 5 supramolecular assemblies. For the computation of the ^1^H and ^13^C shielding tensors, the M06-2X/TZVP level of theory was employed, as implemented in Gaussian 16.^[S24]^ All calculations were implemented with Gauge-Independent Atomic Orbital (GIAO) approach and solvent effects (chloroform) were taken into account using the integral equation formalism (IEF-PCM) in implicit polarizable continuum model, with tetramethylsilane (TMS) shielding corrections applied to both ^1^H and ^13^C shielding tensors to compare with experimental results.^[S25]^ The radii and non-electrostatic terms as implemented in Truhlar’s SMD solvation model was adapted, which corresponds to default values in Gaussian 16. This setup has been successfully used to calculate ^1^H and ^13^C shielding tensors for similar organic systems.^[S26]^

**Validation of the computational approach:**

To validate our assumption of the hydrogen bonded polymer networks as 6-mers, a molecular representation from the adapted molecular structure of a monomer was overlaid with the computational model constructed with the above approach, as shown in Figure S22. The root mean squared displacement (RMSD) values between adapted experimental and computational structures of the hydrogen bonded capsules were calculated using Mercury software, with hydrogen atoms excluded from the RMSD calculations.^[S27]^ There was an excellent agreement between the X-ray diffraction structure and the monomer l-**1**_8,_ retrieved from optimised supramolecular assemblies with an RMSD of 0.12 Å, 0.14 Å and 0.16 Å for no guest, structure with C_60_ and structure with C_70_, respectively. These results support our experimental findings, as the propagation of 1D nanotubular structures is an isodesmic process, as shown by minimal structural RMSD change before (RMSD of 0.15 Å) and after (RMSD of 0.19 Å) guest insertion, as shown in Figure S23.


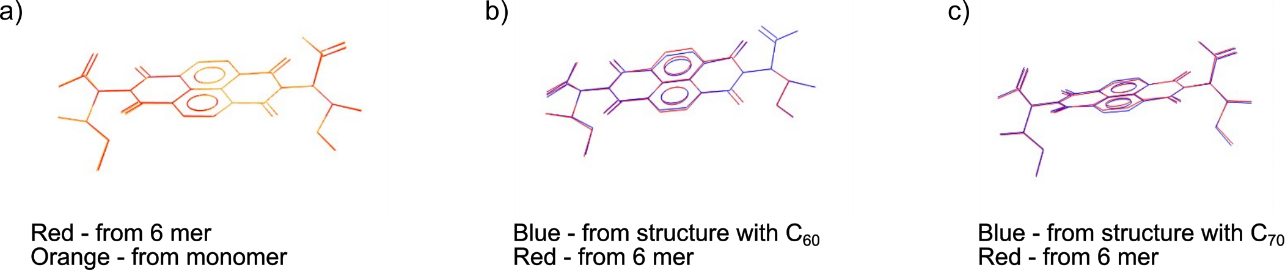


**Figure S22** Overlay of 3D models extracted from DFT calculations a) red – from 6 mer, orange – from monomer; b) red – from 6 mer, blue – from structure with C_60_; c) red – from 6 mer, blue – from structure with C_70_.


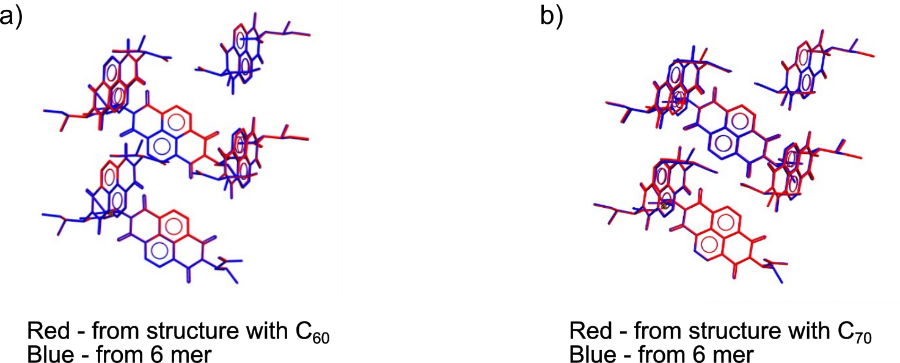


**Figure S23** Overlay of 3D models extracted from DFT calculations a) blue – from 6 mer, red – from structure with C_60_; c) blue – from 6 mer, red – from structure with C_70_.


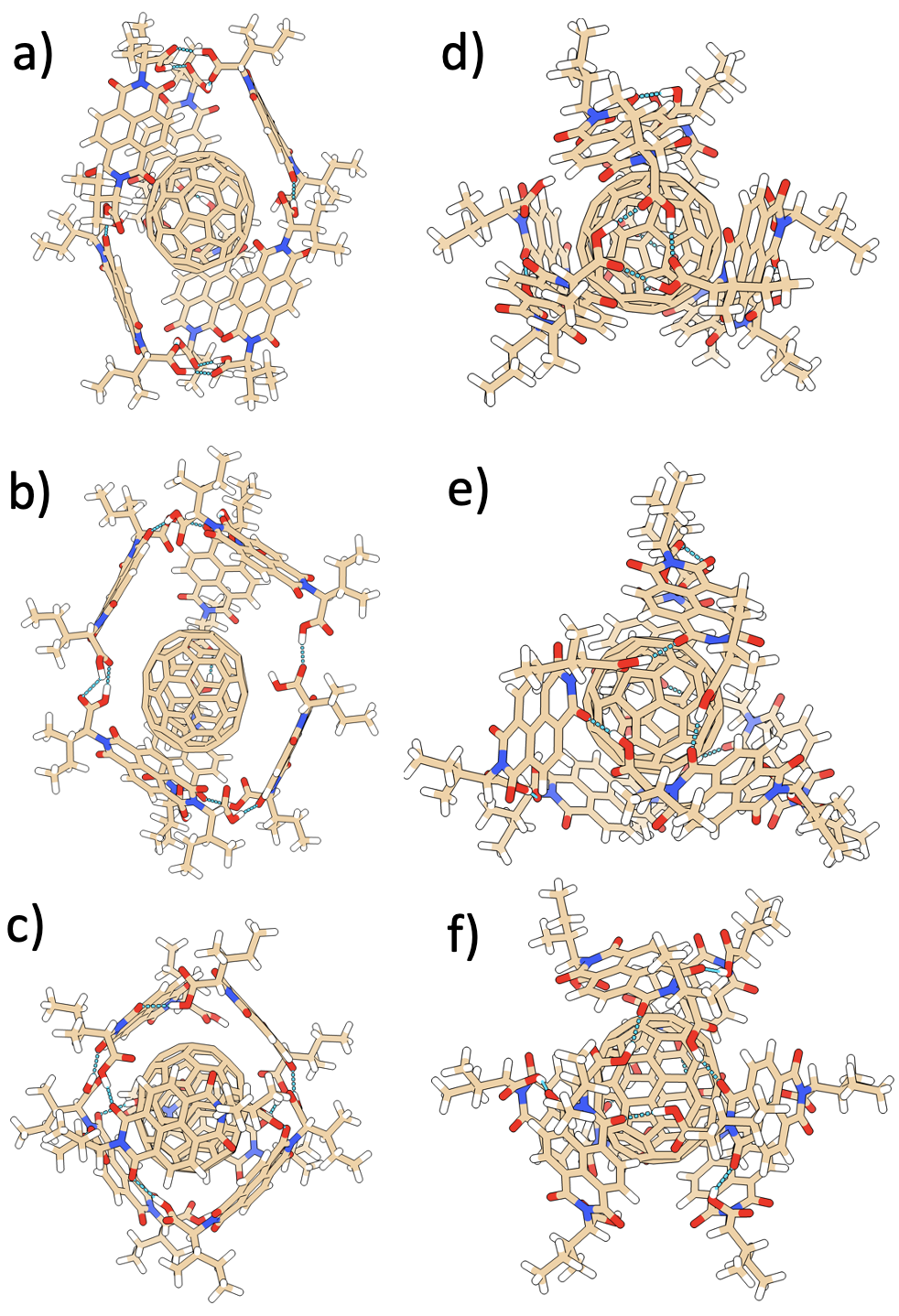


**Figure S24** a) Stick representation for the computational model of Mod. A, Mod. B and Mod. C viewed down the a,b,c) *a*-axis d,e,f) *b*-axis, where *a*-axis highlights top and bottom poles, and *b*-axis the equatorial line. Hydrogen bonding networks are represented by dashed cyan lines. Atoms are colored with respect to hydrogen (white), oxygen (red), carbon (yellow), nitrogen (blue).


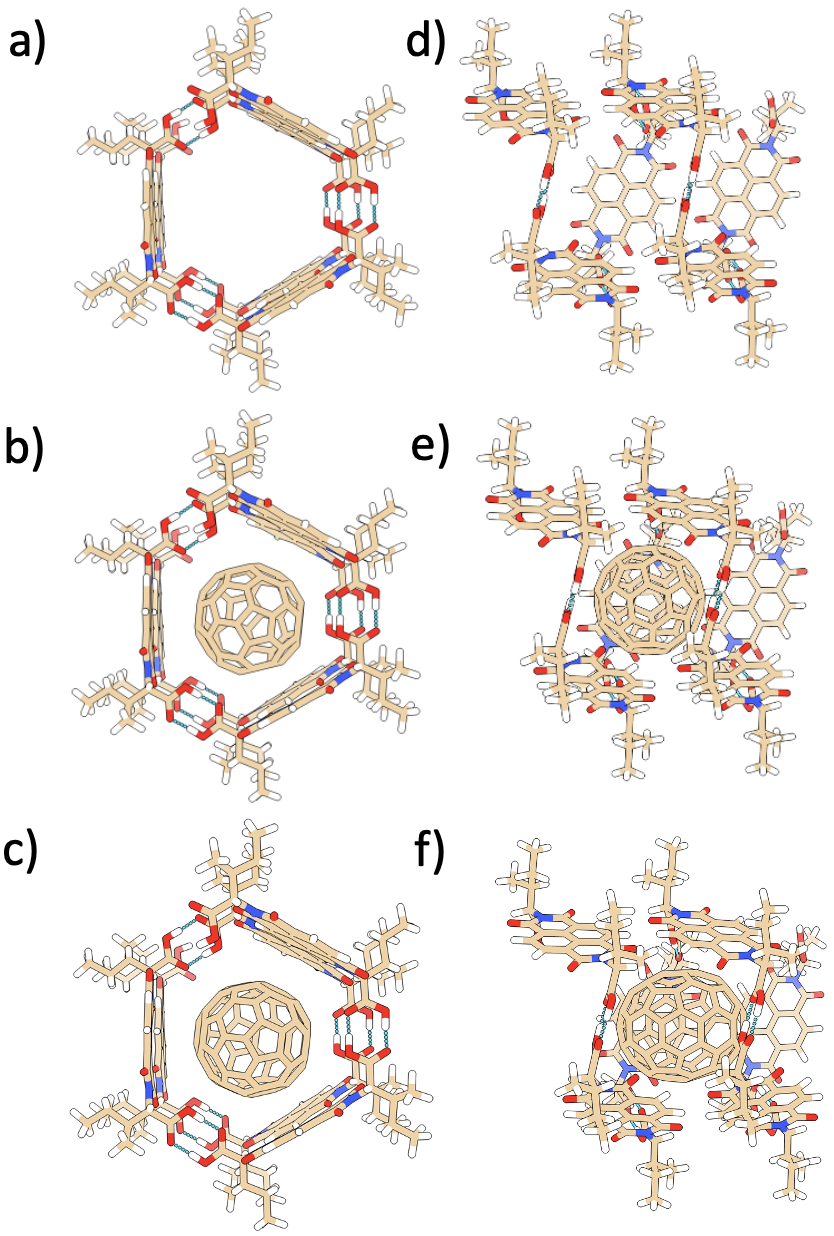


**Figure S25** a) Stick representation for the computational model of Mod. A, Mod. B and Mod. C viewed down the a,b,c) *a*-axis d,e,f) *b*-axis, where *a*-axis highlights top and bottom poles, and *b*-axis the equatorial line. Hydrogen bonding networks are represented by dashed cyan lines. Atoms are colored with respect to hydrogen (white), oxygen (red), carbon (yellow), nitrogen (blue).

Table S2. Calculated DFT energies for the lowest conformation computed for each of the supramolecular assemblies.

| State | DFT Energy (kJ mol^-1^) / PBE/TZVP (D3) |
| --- | --- |
| State 0 | 0 ( Control state) |
| STATE 3 | -42.3 |
| STATE 4a | -70.1 |
| STATE 4b | -190.5 |
| STATE 4c | -220.1 |

Table S3. Calculated DFT energies for the lowest conformation computed for each of the supramolecular capsules.

| STATE 5 modes | DFT Energy (kJ mol^-1^) / PBE/TZVP (D3) |
| --- | --- |
| Mod. A | 0 |
| Mod. B | -65.2 |
| Mod. C | -115.8 |

1. **References**

[S1] Nonius [or Hooft, R. W. W.] (1998). COLLECT. Nonius BV, Delft, The Netherlands.

[S2] Otwinowski Z, Minor W., *Processing of X-ray diffraction data collected in oscillation mode.* Methods Enzymol., 1997, *276*, 307-26.

[S3] Altomare, A., Cascarano, G., Giacovazzo, C., Guagliardi, A., Burla, M. C., Polidori, G. & Camalli, M. *SIR92 - a program for automatic solution of crystal structures by direct methods*. J. Appl. Cryst., 1994, *27*, 435.

[S4] G. Sheldrick, G. M. *Crystal structure refinement with SHELXL*. Section C. Acta. Cryst., 2015, *71*, 3-8.

[S5] Oxford Diffraction, CrysAlis PRO, Oxford Diffraction Ltd, Yarnton, England, 2009.

[S6] Dolomanov, O. V., Bourhis, L. J., Gildea, R. J., Howard, J. A., Puschmann, H. *OLEX2: a complete structure solution, refinement and analysis program*. J. Appl. Crystallogr., 2009, *42*, 339-341.

[S7] Sheldrick, G. M. *SHELXT – Integrated space-group and crystalstructure determination.* Section A. Acta. Cryst., 2015, *71*, 3-8.

[S8] Pantos, G.D., Pengo, P., and Sanders, J.K.M. *Hydrogen-bonded helical organic nanotubes*. Angew. Chem. Int. Ed. 2007, 46, 194-197.

[S9] Wietor, J.L., Pantos, G.D., and Sanders, J.K.M. *Templated amplification of an unexpected receptor for C_70_.* Angew. Chem. Int. Ed. 2008, 47, 2689-2692.

[S10] Stefankiewicz, A.R., Tamanini, E., Pantos, G.D., and Sanders, J.K.M. *Proton-Driven Switching Between Receptors for C(60) and C(70).* Angew. Chem. Int. Ed. 2011, 50, 5724-5727.

[S11] Chen, Z., Lohr, A., Saha-Möller, C. R., Würthner, F. *Self-assembled π-stacks of functional dyes in solution: structural and thermodynamic features*. 2009, Chem. Soc. Rev., *38*, 564-584.

[S12] Ponnuswamy, N., Pantos, G. D., Smulders, M. M. J., Sanders, J. K. M. *Thermodynamics of supramolecular naphthalenediimide nanotube formation: the influence of solvents, side chains, and guest templates.* 2012 J. Am. Chem. Soc., *134*, 566-573.

[S13] Smulders, M. M. J., Nieuwenhuizen, M. M. L., de Greef, T. F. A., van der Schoot, P., Schenning, A. P. H. J., Meijer, E. W. *How to distinguish isodesmic from cooperative supramolecular polymerisation*. Chem. Eur. J., 2010 *16*, 362.

[S14] Markiewicz, G., Smulders, M. M. J., Stefankiewicz, A. R. *Steering the Self-Assembly Outcome of a Single NDI Monomer into Three Morphologically Distinct Supramolecular Assemblies, with Concomitant Change in Supramolecular Polymerization Mechanism*. Adv. Sci., 2019 *6*, 1900577.

[S15] Galaon, T., David, V. *Deviation from van't Hoff dependence in RP-LC induced by tautomeric interconversion observed for four compounds*. J. Sep. Sci., 2011 *34*, 1423–1428.

[S16] L. Turcani, E. Berardo and K. E. Jelfs, J Comput Chem, 2018, 39, 1931–1942.

[S17] Mohamadi, F., Richards, N.G.J., Guida, W.C., Liskamp, R., Lipton, M., Caufield, C., Chang, G., Hendrickson, T. and Still, W.C., *Macromodel—an integrated software system for modeling organic and bioorganic molecules using molecular mechanics*. J. Comput. Chem., 1990 11: 440-467.

[S18] E. F. Pettersen, T. D. Goddard, C. C. Huang, E. C. Meng, G. S. Couch, T. I. Croll, J. H. Morris and T. E. Ferrin, Protein Science, 2021, 30, 70–82.

[S19] T. D. Kühne, *et al.,* *CP2K: An electronic structure and molecular dynamics software package - Quickstep: Efficient and accurate electronic structure calculations,* J. Chem. Phys. 2020, *152*, 194103.

[S20] J. P. w, *et al.,* *Generalized gradient approximation made simple,* *Phys. Rev. Lett.* 1996, *77*, 3865–3868.

[S21] J. VandeVondele, J. Hutter, *Gaussian basis sets for accurate calculations on molecular systems in gas and condensed phases,* *J. Chem. Phys.* 2007*, 127*, 114105.

[S22] S. Grimme, *et al.*, *A consistent and accurate ab initio parametrization of density functional dispersion correction (DFT-D) for the 94 elements H-Pu,* *J. Chem. Phys.* 2010, *132*, 154104

[S23] R. H. Byrd, P. H. Lu, J. Nocedal, C. Y. Zhu, *A Limited Memory Algorithm for Bound Constrained Optimization,* SIAM J. Sci. Comput. 1995, 16, 1190-1208.

[S24] Frisch, M. J.; Trucks, G. W.; Schlegel, H. B.; Scuseria, G. E.; Robb, M. A.; Cheeseman, J. R.; Scalmani, G.; Barone, V.; Petersson, G. A.; Nakatsuji, H.; et al. Gaussian 16 Rev. B.01. Wallingford, CT 2016.

[S25] Marenich, A. V; Cramer, C. J.; Truhlar, D. G. *Universal Solvation Model Based on Solute Electron Density and on a Continuum Model of the Solvent Defined by the Bulk Dielectric Constant and Atomic Surface Tensions*. J. Phys. Chem. B 2009, 113 (18), 6378–6396.

[S26] E. Benassi. *Benchmarking of density functionals for a soft but accurate prediction and assignment of ^1^H and ^13^C NMR chemical shifts in organic and biological molecules*. J. Comput. Chem. 2017, 38, 87–92.

[S27] C. F. Macrae, I. Sovago, S. J. Cottrell, P. T. A. Galek, P. McCabe, E. Pidcock, M. Platings, G. P. Shields, J. S. Stevens, M. Towler and P. A. Wood, J Appl Crystallogr, 2020, 53, 226–235.
